# Range-wide parallel climate-associated genomic clines in Atlantic salmon

**DOI:** 10.1098/rsos.171394

**Published:** 2017-11-15

**Authors:** Nicholas W. Jeffery, Ryan R. E. Stanley, Brendan F. Wringe, Javier Guijarro-Sabaniel, Vincent Bourret, Louis Bernatchez, Paul Bentzen, Robert G. Beiko, John Gilbey, Marie Clément, Ian R. Bradbury

**Affiliations:** 1 Fisheries and Oceans Canada, Northwest Atlantic Fisheries Centre, St John's, Newfoundland and Labrador, Canada A1C 5X1; 2 Faculty of Computer Science, Dalhousie University, Halifax, Nova Scotia, Canada B3H 4R2; 3 Department of Biology, Dalhousie University, Halifax, Nova Scotia, Canada B3H 4R2; 4 Fisheries and Oceans Canada, Bedford Institute of Oceanography, Dartmouth, Nova Scotia, Canada B2Y 4A2; 5 Laboratoire d'expertise biolégale, MFFP, Québec, Québec, Canada G1P 3W8; 6 Department of Biology, Université Laval, Québec, Québec, Canada G1 V 0A6; 7 Marine Scotland, Freshwater Fisheries Laboratory, Faskally, Pitlochry PH16 5LB, UK; 8 Centre for Fisheries Ecosystems Research, Fisheries and Marine Institute, Memorial University of Newfoundland, St John's, NL, Canada; 9 Labrador Institute, Memorial University of Newfoundland, Happy Valley-Goose Bay, NL, Canada

**Keywords:** Atlantic salmon, SNPs, clines, adaptation, parallel evolution

## Abstract

Clinal variation across replicated environmental gradients can reveal evidence of local adaptation, providing insight into the demographic and evolutionary processes that shape intraspecific diversity. Using 1773 genome-wide single nucleotide polymorphisms we evaluated latitudinal variation in allele frequency for 134 populations of North American and European Atlantic salmon (*Salmo salar*). We detected 84 (4.74%) and 195 (11%) loci showing clinal patterns in North America and Europe, respectively, with 12 clinal loci in common between continents. Clinal single nucleotide polymorphisms were evenly distributed across the salmon genome and logistic regression revealed significant associations with latitude and seasonal temperatures, particularly average spring temperature in both continents. Loci displaying parallel clines were associated with several metabolic and immune functions, suggesting a potential basis for climate-associated adaptive differentiation. These climate-based clines collectively suggest evidence of large-scale environmental associated differences on either side of the North Atlantic. Our results support patterns of parallel evolution on both sides of the North Atlantic, with evidence of both similar and divergent underlying genetic architecture. The identification of climate-associated genomic clines illuminates the role of selection and demographic processes on intraspecific diversity in this species and provides a context in which to evaluate the impacts of climate change.

## 1. Introduction

The analysis of geographically based genomic clines can illuminate the causes of population differentiation and reveal the interaction between natural selection and dispersal. Clinal variation has frequently been linked to biotic and abiotic factors, notably environmental gradients (e.g. temperature [1]; salinity [2–4]), which can impose spatially differential selective pressures. There is growing evidence of environmentally influenced variation in allele frequency, documented through significant correlations with latitude and environment factors (e.g. seasonal temperature ranges). Regional differences in selective pressure may, therefore, be strong enough to overcome gene flow or drift among geographically distinct populations. For example, clines in *Drosophila melanogaster* alcohol dehydrogenase (*Adh*) have been revealed across different continents both historically [5,6] and as a response to climate change [7]. Because clines can form and vary in response to environmental selection, their location and intensity may shift with climate change. Ultimately, the identification of climate-associated genomic clines can illuminate the role of evolutionary processes on intraspecific diversity and provide a context against which to evaluate climate change impacts.

Selection is often implicated as a cause of climate-associated clines in allele frequency, although it can be challenging to disentangle it from other demographic influences. One approach is to focus on parallel clinal variation along replicated environmental gradients, which when present, can provide some of the strongest evidence for adaptive divergence under natural conditions [8]. Schmidt *et al*. [9] suggest the natural replication of environmental gradients in the North Atlantic along the North American and European coasts is ideal for testing parallel adaptation in marine organisms. Parallel phenotypic adaptation can occur with either similar [1,10] or different [11–13] underlying genetic architecture, and polygenic traits may not necessarily show clinal patterns in all loci contributing to that trait [14,15]. Rapid advances in sequencing technologies provide the ability to genotype thousands of single nucleotide polymorphisms (SNPs) from coding and non-coding regions of the genome [16] providing large numbers of candidate loci for studying clinal patterns at a number of scales [9,14,17]. For example, Bradbury *et al*. [1] revealed parallel adaptation to ocean temperature in Atlantic cod (*Gadus morhua*) in the eastern and western North Atlantic, which has recently been shown to be associated with shared inversions on multiple chromosomes [18,19]. The broad distribution of species on both the eastern and western North Atlantic, and the ability to genotype individuals with high-throughput sequencing, provides a compelling experimental ground to test for parallel adaptation in a naturally replicated environment.

Atlantic salmon (*Salmo salar*) are naturally distributed across approximately 30° latitude on both sides of the North Atlantic [20]. Eastern and western lineages display significant isolation evidenced by clear genetic and genomic divergence [21] and differences in chromosome number and structure [22,23]. Divergence between North American and European salmon occurred *ca* 600 000 years ago (600 kA) [24], although much of the contemporary range was only recently recolonized (approx. 10 kA) following the last glacial maximum [25]. Within lineages, however, fine-scale patterns of genetic structure have been observed with high-density SNP arrays, and population structure is evident among regions, rivers, and even tributaries within a river system [26–29] and appears to be significantly associated with the environment [30]. Latitudinal variation in life history, including age at first seaward migration (smolt age) [31], spawning age [32] and number of winters spent at sea [25] has been well described in both the eastern and western Atlantic ranges. To date, observations of genetic clines in Atlantic salmon have been limited to the detection of multilocus clines in allele frequency with coastal distance in European populations, where it is unknown whether secondary contact associated with reproductive barriers or true latitudinal local adaptation is occurring in the Baltic and Barents Seas [21]. Dionne *et al*. [33] revealed correlations between latitude and temperature for major histocompatibility complex alleles in Canadian rivers potentially due to a decrease in bacterial or ectoparasite diversity with latitude [34]. Genetic evidence of parallel adaptation between European and North American populations is suggested by a single study, Verspoor & Jordan [35], who revealed significant associations in the *Me-2* locus allele frequencies with latitude and summer temperature. As such, congruence in clinal variation in the eastern and western Atlantic and the associated genomic architecture for Atlantic salmon remains largely unknown.

Here we investigate range-wide clines in allele frequency with latitude and environmental variables using SNP genotype data in North American and European wild salmon populations. Specifically, we aim to test whether divergent or parallel evolution is occurring in the Atlantic Ocean by first comparing which loci show clinal patterns in North America and Europe, and then investigating how the spatial structure of clinal loci varies with latitude, temperature and geographic distances. This study will help elucidate whether North American and European lineages have responded similarly to climate-associated selection, and more generally will contribute to the understanding of evolution in parallel, naturally replicated environments. We build directly on previous studies documenting range-wide genomic variation in Atlantic salmon [21,26,36] and use available SNP data [21,37] for Atlantic salmon from throughout the North Atlantic.

## 2. Material and methods

### 2.1. Combined datasets

To maximize the number of sampling locations we combined publicly available genotype data, including high-resolution North American data from Moore *et al*. [26] and [36], European and North American data from Bourret *et al*. [21], recent data from Lake Melville and Labrador [38] and United Kingdom river data from Gilbey *et al*. [37] (electronic supplementary material, table S1). All datasets were filtered for monomorphic loci and minor allele frequencies less than 0.01 using PLINK [39]. We also evaluated SNPs missing data in greater than 15% of individuals and removed these. We filtered the North American and European datasets to 1773 loci common to all locations and SNP arrays using the R package *genepopedit* [40]. While we note that our European data are unbalanced due to the inclusion of a high density of northern English and Scottish rivers, we investigated whether random subsetting these rivers down to four populations would alter our results, and they did not noticeably change our detected clinal loci or population structure results. This is perhaps because these rivers do not vary by latitude to a large degree (less than 4°), and only represent environmental data from four different weather stations in this region. Thus we included all rivers in our analyses to make this combined dataset publicly available. Summary statistics, including observed heterozygosity and locus-specific *F*_ST_ values were calculated in the R package *hierfstat* [41]. Following SNP filtering, we briefly investigated population structure using AMOVA, isolation-by-distance, principal components analysis and a neighbour-joining (NJ) tree (see electronic supplementary material, methods).

We tested for outlier loci from this panel of 1773 loci using two methods, splitting our data into separate continents to account for potentially different loci being under selection. First, we used BayeScan v. 2.1 [42], a Bayesian method that identifies outlier loci using differences in allele frequency among populations which accounts for differences in sample size, with prior odds of 100 and 5000 Markov chain Monte Carlo (MCMC) iterations. We then implemented a hierarchical island model for outlier detection in ARLEQUIN 3.5.2.2 [43,44] with 20 000 simulations, 100 demes per group and 30 groups to simulate both the North American and European datasets. This method was selected to supplement BayeScan to control for local hierarchical structure, and thus select only broad-scale potential outliers [44].

### 2.2. Clinal loci and analysis

To detect loci showing clinal patterns in allele frequency, we used binomial logistic regression of allele frequencies against latitude on all 1773 loci separately in Europe and North America. Any locus which showed a sigmoidal or exponential pattern and exponential decay at each respective tail end of its distribution [45,46] with a steep change in allele frequency (greater than 40%) across its latitudinal range was deemed clinal in nature. This 40% threshold was chosen based on a sharp decline in the number of loci selected at a range of thresholds based on the empirical cumulative distribution function in the R stats package. These loci were subset from our full SNP panels using genepopedit [40] to create clinal North American and European datasets. We visualized the genomic distribution of clinal loci as Manhattan plots using locus-specific *F*_ST_ aligned to genomic positions from linkage maps generated for North America [23] and Europe [47].

Environmental data, including annual and seasonal temperatures, annual rainfall and snowfall, number of days with temperature greater than 0°C and 10°C, as well as degree days greater than 0°C and 10°C was collated for North America using climate archives from weather stations closest to each river averaged from 1981 to 2010 (http://climate.weather.gc.ca/climate_normals/) (electronic supplementary material, table S2). Average annual and seasonal temperatures and average precipitation data were collected from the nearest weather stations for European rivers (http://www.climatedata.eu/) (electronic supplementary material, table S2).

We used multiple regression to determine the environmental variable(s) that best correlated with latitude, and accounted for collinearity among variables by minimizing their variance inflation factors below 5. We supplemented this analysis with redundancy analyses (RDA), which were used to select the environmental predictor(s) which best explained genotype variation in each continent. For the RDA we used the allele frequencies of clinal loci as a multivariate-dependent variable representing individual genotypes and all of the environmental variables as the explanatory variables, both with and without controlling for latitude and longitude. Overall RDA significance was assessed using a permutation test with 1000 permutations, while the most significant variables were determined using a type-III ANOVA with 1000 permutations.

We then evaluated clinal structure using STRUCTURE [48] admixture coefficients and discriminant analysis of principal components (DAPC) membership probabilities [49] assuming *K*, the putative number of populations, was equal to two, to characterize a latitudinal divide. To incorporate spatial information directly when characterizing differentiation between northern and southern groupings, we also performed a spatial principal components analysis (SPCA) [49] using a Delaunay spatial proximity network [50]. SPCA incorporates spatial variability directly by accounting for spatial autocorrelation among individuals or populations when optimizing the variance of principal components. Spatial relationships between stations were characterized using a custom R function [51], which incorporates least-cost distances among the mouths of each river within each continent separately using *marmap* [52] and re-projects them as Cartesian coordinates (electronic supplementary material, figure S1), accounting for land as an impermeable barrier to movement, using non-metric multidimensional scaling in *vegan* [53]. From the SPCA analyses we retained the first lagged axis scores to characterize the northern and southern delineation.

We standardized output from each clustering method according to latitude, south to north (0–1). SPCA lagged axis scores were standardized in the same 0–1 scale for comparison with output from STRUCTURE and DAPC. We then separately modelled each coefficient as a function of latitude, distance from the southernmost site, and environmental parameters using generalized logistic regressions. Environmental variables used in this analysis were selected by RDA, highlighting the most significant relationships between clinal structure and climate. The significance, Akaike information criterion (AIC) and McFadden's pseudo-*r*^2^ [54] are reported for each model. Because our European data has a high proportion of United Kingdom rivers, we ran the same test twice with all rivers and with a random subsample of four Scottish rivers to test for biases in the dataset.

Finally, to account for different phylogeographic groups in the data leading to the observed structure by chance or neutral processes, we removed the Baltic rivers from the European data, as these are known as a distinct evolutionary lineage [55] and ran the same logistic regression model as above. We additionally attempted to account for neutral processes by using a partial Mantel test based on dissimilarity matrices of *F*_ST_ between populations using the clinal SNPs, winter and spring temperature matrices, and controlling for geographic distance among populations using 999 permutations in the *vegan* package.

### 2.3. Clinal SNP gene associations

We implemented BLAST searches of approximately 400–2000 bp of flanking sequence for each of the 12 SNPs that showed clinal patterns in both North America and Europe with a minimum identity of 98% and *E*-values less than 0.0001 to determine which genes, if any, are associated with these loci and their putative functions.

## 3. Results

### 3.1. Locus filtering and outlier detection

**Figure 1.**
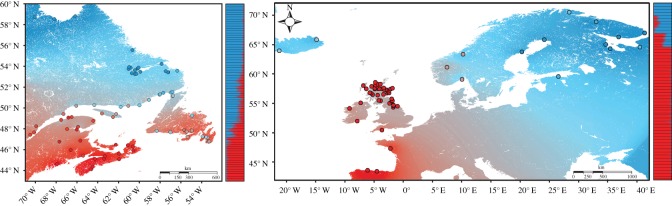
Maps showing all sampling locations across North America and Europe. Details for each river can be found in electronic supplementary material, table S1. The average annual air temperature is shown as an interpolated gradient across North America and Europe, while points are shaded by their STRUCTURE admixture coefficients assuming *K* = 2 within each continent. Both temperature and *Q*-values scale from 0 to 1, where dark red = 0 and dark blue = 1. Bar plots beside each map represent individual admixture coefficients for each population within each continent.

There were 2759 loci in common to the CIGENE 6 K and 220 K Affymetrix SNP chips. After combining and filtering data from Bourret *et al*. [21] and Gilbey *et al*. [37], we were able to construct a dataset of 1852 loci that were common to all datasets. Filtering of this common dataset for minor allele frequency and removal of monomorphic loci resulted in 1773 loci common to all populations. This final dataset included 3942 individual salmon from 74 North American and 60 European wild populations, covering a range of approximately 15° and 27° latitude, respectively (figure 1; electronic supplementary material, table S1). The average global *F*_ST_ across all 1773 loci for North America was 0.110 and 0.089 for Europe, while average observed heterozygosity was substantially lower in North America (0.134) relative to Europe (0.337). In North America, 62 loci (3.5%) were detected as outliers by both BayeScan and Arlequin, while only 19 loci (1.0%) were detected as outliers in Europe. Only two loci (ESTNV_34251_1165 and GCR_cBin48984_Ctg1_170) were revealed as outliers in both North America and Europe.

### 3.2. Population structure

An AMOVA revealed a high degree of genetic differentiation between North American and European populations (*F*_CT_ = 0.369), as well as significant variance among populations within groups (*F*_ST_ = 0.427; electronic supplementary material, table S3). Both North America and Europe showed significant positive relationships between linearized *F*_ST_ and least-cost geographic distance (*r*^2^ = 0.249, *p* < 0.0001 and *r*^2^ = 0.621, *p* < 0.0001, respectively; electronic supplementary material, figure S2). Principal component analysis clearly resolved North American and European rivers along the first principal component axis (electronic supplementary material, figure S3), and the NJ tree revealed that individual North American and European rivers clustered consistently among northern and southern groups, agreeing with previously published microsatellite and SNP data [21,26,36] (electronic supplementary material, figure S4).

### 3.3. Clines in allele frequency

**Figure 2.**
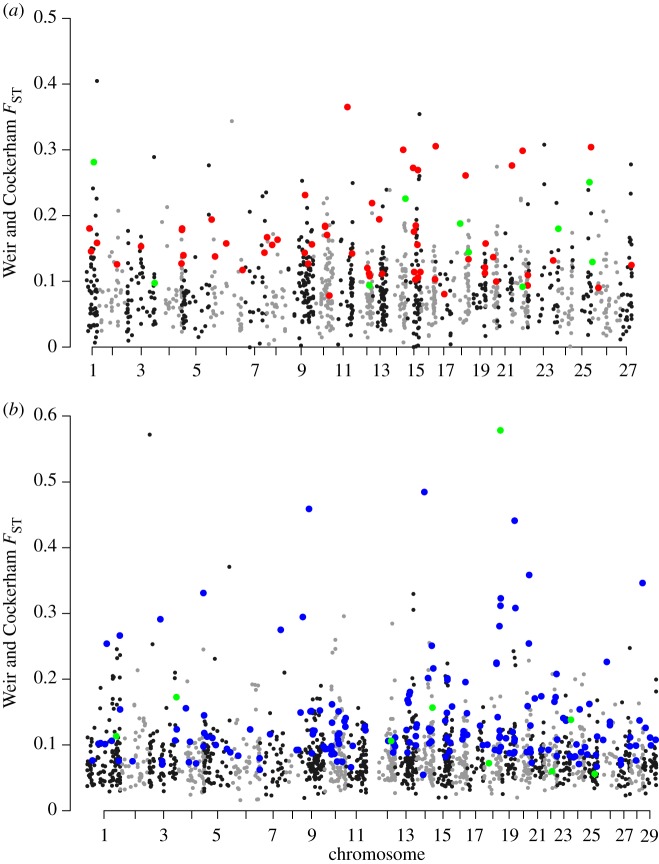
Manhattan plots of locus-specific *F*_ST_ versus genomic position for (*a*) North American clinal loci (red), (*b*) European clinal loci (blue) and loci that were clinal in both continents (green). Linkage maps from Brenna-Hansen *et al*. [23] and Lien *et al*. [47] were used to obtain North American and European positions, respectively.

We detected 84 (4.7%) North American and 195 (11%) European loci showing clinal patterns across their respective latitudinal ranges (electronic supplementary material, figure S5) and 12 of these overlapped between continents. Using a permutation test with 100 000 sampling iterations we calculate that the probability of detecting 12 loci in common based on 84 and 195 loci from each continent to be 0.11, suggesting that this is a rare phenomenon not necessarily due to chance alone. *F*_ST_ values for North American clinal loci ranged from 0.078 to 0.54 $\left(\bar{x}=0.167\right)$, and ranged from 0.053 to 0.58 $\left(\bar{x}=0.127\right)$ in Europe. These clinal loci were evenly distributed across the genomes of both North American and European salmon (figure 2). In Europe, 15 of these clinal loci were detected as outliers by BayeScan and Arlequin, while 18 clinal loci were detected as outliers in North America.

In North America, average annual and seasonal temperatures and degree day data were highly correlated with latitude, with average annual temperature showing the highest collinearity among environmental variables. Multiple regression analysis, controlling for this multicollinearity by removing annual average temperature, revealed that average winter temperature (*p* < 0.0005) and the number of degree days exceeding 10°C (*p* < 0.0005) were the most highly correlated with latitude (electronic supplementary material, figure S6). This significance remained even after removing the three high-latitude rivers (Ungava), which appear to anchor the relationship (electronic supplementary material, figure S6). In Europe, average annual temperature was again highly correlated with seasonal temperature data, and after removal of this parameter from a multiple regression model, average autumn and winter temperature remained the most significantly correlated with latitude (*p* < 0.0001; electronic supplementary material, figure S7). RDA of the environmental data and clinal membership allele frequencies were globally significant for North America (*F *= 15.46, *p* < 0.001) and Europe (*F *= 152.77, *p* < 0.001) (electronic supplementary material, figure S8). A type-III ANOVA revealed that winter seasonal temperature and annual snowfall were the two variables most associated with clinal structure (*F *= 9.68, *p* < 0.001 and *F *= 17.74, *p* < 0.001, respectively) in North America, whereas seasonal winter, spring and summer temperatures were the most significantly associated with clinal structure in Europe (*F *= 38.89, *p* < 0.001; *F *= 95.38, *p* < 0.001; and *F *= 96.48, *p* < 0.001, respectively).

**Figure 3.**
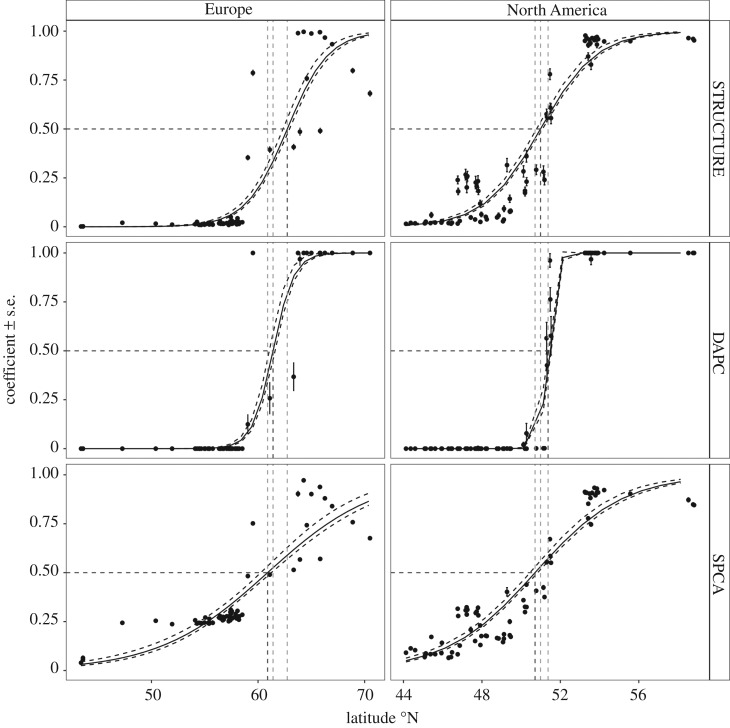
Spatial clustering analysis of admixture coefficients (STRUCTURE), membership probabilities (DAPC), and normalized first axis lagged scores (SPCA) based on clinal loci with latitude within Europe and North America. Vertical dashed lines represent the predicted inflection points for each tested method. Generalized logistic model fits are presented with their standard errors (dashed lines).

**Figure 4.**
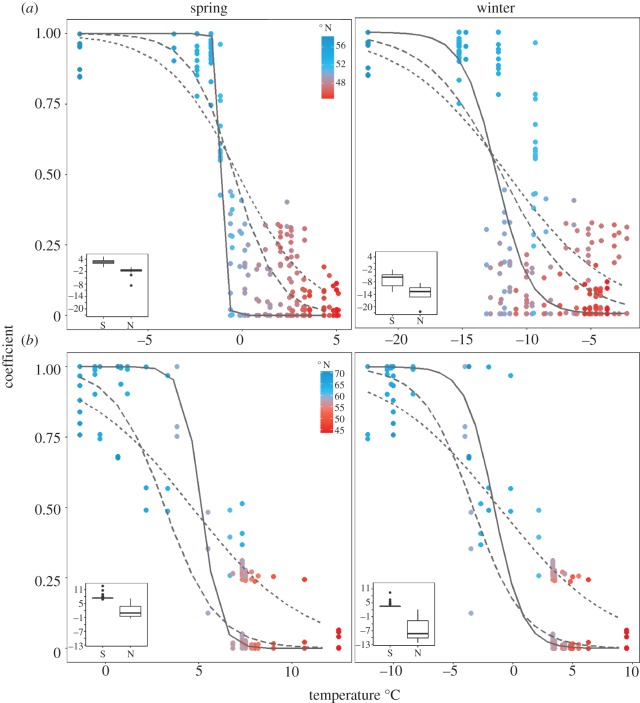
Generalized logistic regression of combined coefficients from STRUCTURE (short dashed line), DAPC (solid line), and SPCA (long dashed line) with average spring temperature and average winter temperature for (*a*) North America and (*b*) Europe. Inset boxplots show the range and median temperatures (°C) for rivers categorized as northern or southern by the analyses. Points are coloured by latitude, with red corresponding to low latitudes and blue corresponding to high latitudes.

DAPC, STRUCTURE and SPCA analyses using only clinal loci highlighted a latitudinal divide in populations within each continent (figure 3; electronic supplementary material, figure S9). Generalized logistic regressions of the respective clustering coefficients from each analysis showed significant relationships with latitude, distance, annual and seasonal temperatures, degree days and precipitation (*p* < 0.0001 in all cases, see electronic supplementary material, table S4 for summary statistics). In North America, the predicted inflection point among northern and southern populations was at 53.3°N in southern Labrador (figure 1; electronic supplementary material, figure S5), and clinal clustering coefficients were strongly correlated with spring temperatures (*r*^2^ = 0.82, figure 4*a*). In Europe, winter and spring temperature showed the strongest relationships with clinal clustering coefficients (*r*^2^ = 0.89 for each, figure 4*b*), and the predicted latitudinal inflection point was 59.9° N, corresponding to southern Norway (figure 1; electronic supplementary material, figure S5).

Significance of winter and spring temperatures did not change after subsetting a random sample of United Kingdom rivers to control for bias, or removal of the Baltic rivers from the European data. Results from a partial Mantel test controlling for geographic distance still showed significant relationships between differences in population *F*_ST_ values and winter and spring temperature for North America (*r* = 0.28, *p* = 0.001; *r* = 0.28, *p* = 0.001, respectively) and Europe (*r* = 0.31, *p* = 0.001; *r* = 0.26, *p* = 0.007, respectively).

### 3.4. SNP annotation

Of the 12 loci that showed clinal patterns in both continents, nine were found to be in close proximity to genes based on their flanking sequences. The putative functions of these loci include roles in development (retinoic acid receptor γ*b*), solute (potassium and chloride) transport, transcription factors and the regulation of cytokinesis (*prc1* gene) (electronic supplementary material, table S5).

## 4. Discussion

Spatial clines in allele frequency across repeated environmental gradients can provide insight into the demographic and evolutionary processes that shape intraspecific diversity [56]. Here we report genomic clines in allele frequency associated with climate in both North American and European Atlantic salmon populations, suggesting parallel climate-associated evolution occurring in similar North Atlantic coastal habitats as a result of adaptive diversity or historical demographic processes. We build directly on previous studies indicating fine-scale regional structure in both Europe and North America using allozymes [29], microsatellites [25] and SNPs [21,26] and recent work reconstructing demographic influences on contemporary diversity [57]. Using clinal clustering coefficients, we detect that the observed clinal structure strongly correlates with latitude and spring temperature in North America and Europe. This observed climate-associated genomic structure will inform management efforts by incorporating population structure directly and the knowledge that different populations will probably respond differently to climate change into conservation planning, and allow the assessment and prediction of climate change impacts in Atlantic salmon.

We identified approximately 5% and 11% of loci which showed clinal patterns in North America and Europe, respectively which were evenly distributed across the genome and had higher *F*_ST_ values than non-clinal loci, suggesting potential genome-wide climate-associated adaptation. While this discrepancy in the number of clinal loci detected between continents and the lower heterozygosity of the North American SNPs may be the result of ascertainment bias from the design of this SNP array [21], the fact that we selected clinal loci independently between continents should remove this bias for the purpose of studying latitudinal patterns. No relationship between latitude and heterozygosity within continents was detected, reinforcing that the observed clinal patterns are not simply due to demographically associated losses of heterozygosity at high latitudes. Both continents showed significant relationships between clinal structure and spring temperature, and while rainfall and snowfall also showed significant relationships, these explained the least of the variation. Spring temperature, coinciding with increased snowmelt and river discharge, has previously been found to explain annual smolt run timing in some populations [58], and is also a time of growth and feeding following the colder winter temperatures [59]. Spring temperatures also correspond with the timing of adult return migrations to their natal rivers in some populations prior to spawning, with earlier migrations triggered by higher spring temperatures [59]. As such, spring temperatures appear to be critical at various life stages of Atlantic salmon and thus can exert selective pressures for local adaptation along a latitudinal gradient.

Our results suggest parallel patterns of diversity of warm- and cold-water adapted or acclimated populations based on both divergent and parallel genetic architectures between continents, as 12 loci showed clinal patterns in both continents, while the remainder of the clinal loci were unique to either North America or Europe. These common clinal loci support a hypothesis of climate-associated adaptive evolution despite different demographic histories in the North Atlantic [9]. Putative functions of these loci include cell division, growth and development and solute transport, reinforcing the evidence that clinal variation with spring temperatures may show local adaptation as a result of differences in fry and smolt growth rates, or adaptations to varying physical properties of their natal rivers. This parallel evolution with a complex underlying genetic architecture is possible due to different mutations within a gene with similar phenotypic effects, mutations in different genes that result in the same phenotype, and pleiotropic effects among genes [14,15]. Similar results of parallel phenotypic evolution with different genetic bases have been detected for predation regimes in guppies [13], freshwater versus marine adaptation in stickleback [60] and opsin expression in cichlids [12]. This pattern is in stark contrast to Atlantic cod, which show parallel temperature adaptation at the same loci in the east and west North Atlantic [1]. However, North American and European Atlantic salmon diverged *ca* 600 kA [24], allowing for ample time for genomic divergence while still showing temperature-associated adaptations in parallel North Atlantic environments.

Previous work by Bourret *et al*. [21] detected clinal patterns in allele frequency in European populations, and found that European clinal loci were randomly distributed across the genome, while environmentally associated outliers are also randomly distributed across the genome in North America [30], similar to our results presented here. Dionne *et al*. [61], previously showed correlations between major histocompatibility allelic richness with temperature, degree days and bacterial diversity in a preliminary analysis of how different North American populations are differentially adapted along a cline. Verspoor & Jordan [35], in the first study to investigate parallel genetic evolution in salmon between continents, showed a significant relationship between allelic variation in the *Me-2* locus and summer temperatures in both North America and Europe, which suggest at least some degree of similar underlying genetic architecture leading to parallel evolution in Atlantic salmon. By contrast, none of the SNP annotations we conducted revealed an association with the location of this gene and so we could not corroborate this evidence.

While these correlations with temperature may represent adaptive differences, as we found that spring and winter temperatures were still significantly associated with genetic structure even after controlling for the isolation-by-distance relationship and removal of the Baltic phylogroup, we suggest that it is a combination of adaptive and neutral processes over geological time which has led to this observed clinal structure. In addition to being driven by local adaptation, the observed clines are probably influenced by demographic processes including secondary contact among populations and barriers to gene flow, or a combination of these. In *Drosophila* for example, both secondary contact and local adaptation have contributed to inter-continental parallel clinal patterns [6]. Recent evidence suggests genetic incompatibilities among populations may be more commonly involved in forming clinal patterns than contemporary environmental associated adaptation [62]. In Atlantic salmon, demographic processes following the most recent glaciation, including extensive periods of allopatry in glacial refugia [63] and more recent secondary contact could allow for genetic incompatibilities limiting gene flow and introgression [28,57]. An in-depth analysis of inter- and intra-continental demographics by Rougemont & Bernatchez [57] suggests that following the last glacial maximum, salmon in southern East Atlantic refugia probably colonized northern Europe over time, and more recently, North America was colonized by multiple European sources *ca* 13 kA. Subsequent secondary contact both within and between continents is suggested to have eroded genetic differentiation, unless prevented by barriers to gene flow, or in our case, potentially due to climate-associated selection. Delimiting the relative roles of secondary contact, local adaptation and genomic architecture [18,64] will provide a more complete understanding of the processes that lead to latitudinal clines [57].

Regardless of the processes that ultimately formed these clinal patterns, the impacts of climate change will probably differ significantly among populations across their range. It has been predicted that as temperatures warm, there will be a general northward shift of Atlantic salmon populations, and the poleward shift of marine species in general [65], with a high likelihood of extinction in the southern part of their distribution ([59] and references therein). Climate change is predicted to lead to earlier egg hatching and alevin emergence, as well as earlier smolt migrations [59]. Elliott & Elliott [66] predict that winter and spring water temperatures would need to increase by at least 4°C before growth is negatively affected in brown trout (*Salmo trutta*), a species with narrower temperature tolerances than *S. salar.* As such, it is likely that a northward shift in range of southern populations will occur in the North Atlantic as the climate warms, and northern populations may move northward if habitat exists, or perhaps coexist with individuals from the south. The observed genomic clines provide a means of both modelling climate responses and monitoring ongoing changes in intraspecific diversity.

Admittedly, our study includes relatively few markers (less than 2000) in order to maximize the number of sample geographic locations included. Larger genomic panels are regularly screened in the field of salmonid genomics; however, the goal of our study was to detect clinal patterns along a continuous latitudinal range, and we do not believe that a lack of markers will bias these results. Additionally, we recognize that our European sampling is biased towards United Kingdom rivers, though we do not believe this influences our results due to the relatively similar climate these populations experience and the small differences in latitude between rivers. However, increasing the resolution of European samples is a priority and may help to understand the demographic processes leading to the observed clinal population structure. Including more markers and populations would probably lead to the discovery of additional clinal markers and increase our genomic resolution of these clinal patterns.

## 5. Conclusion

Here we find evidence for climate-associated parallel clinal structure in Atlantic salmon in the east and west North Atlantic Ocean. Genotypes based on clinal loci were significantly associated with spring seasonal temperatures in both continents, and these loci were associated with various functions, including solute transport, development, metabolic and growth genes. Our results can provide useful information for future work projecting the effects of climate change on different populations at different latitudes, and possible overall northward shift of present-day populations. Collectively, this work punctuates the need to design conservation measures which reflect the fine-scale patterns of genetic diversity in Atlantic salmon to ensure the perseverance of regional populations under a changing climate.

## Supplementary Material

Supplementary Methods, Tables, and Figures

## Data accessibility

All data used in the present study is publicly available and cited in the text. Filtered genotypes, R scripts, and metadata used in the analyses are available from the Dryad Digital Repository: https://doi.org/10.5061/dryad.cv20d [67].
