# Supplementary material for: Range-wide parallel climate-associated genomic clines in Atlantic salmon
Source: R Soc Open Sci. 2017 Nov 15;4(11):171394. doi: 10.1098/rsos.171394 (PMC5717698; doi:10.1098/rsos.171394)
Supplement: Supplementary Methods, Tables, and Figures [file rsos171394supp1.docx]

# Supplementary Methods

## Population Structure

We investigated both inter- and intracontinental spatial structure using all 1773 loci and all 134 rivers. We first conducted an analysis of molecular variance (AMOVA), comparing eastern and western Atlantic rivers to evaluate broad-scale continental molecular variation. We then examined the isolation-by-distance (IBD) relationship within each continent between Slatkin’s [1] linearized *F*_ST_ calculated in ARLEQUIN 3.5.2.2 [2] using all 1773 common loci versus least-cost geographic distance among sites calculated in *marmap* [3]. Significance of IBD relationships were evaluated using Mantel tests with 1000 permutations were calculated for each continent in the R package *ade4* [4].

We then investigated individual-river spatial structure using two independent methods. First, we conducted principal component analyses on all rivers using *adegenet* 2.0.1 [5, 6], retaining five eigenvectors and plotting the rivers along the first and second principal components. Second, we constructed a neighbour-joining (NJ) tree based on Cavalli-Sforza and Edwards [7] chord distances with 1000 bootstraps on individuals in POPULATIONS 1.2.33 [8] to evaluate the genetic relationships among rivers. NJ trees were visualized and mid-point rooted in Figtree 1.4.2 (<http://tree.bio.ed.ac.uk/software/figtree/>).

# Supplementary Tables and Figures

**Table S1.** Population names, codes, sample sizes, and geographic coordinates for 134 North American and European populations used in the present study. See the manuscript for references.

| **Site** | **Code** | **n** | **Degrees longitude** | **Degrees latitude** | **Region** | **Data Source** |
| --- | --- | --- | --- | --- | --- | --- |
| Miramichi | MIR | 25 | -65.67 | 46.87 | Gulf of St. Lawrence | Moore et al. [26],  Bradbury et al. [36],  Bourret et al. [21] |
| Antigonish West | ANW | 20 | -61.96 | 45.62 | Gulf of St. Lawrence | Moore et al. [26],  Bradbury et al. [36],  Bourret et al. [21] |
| North | NRV | 20 | -60.62 | 46.3 | Gulf of St. Lawrence | Moore et al. [26],  Bradbury et al. [36],  Bourret et al. [21] |
| Eskasoni | ESK | 20 | -60.6 | 45.94 | Gulf of St. Lawrence | Moore et al. [26],  Bradbury et al. [36],  Bourret et al. [21] |
| Cross | CRO | 20 | -62.26 | 46.48 | Gulf of St. Lawrence | Moore et al. [26],  Bradbury et al. [36],  Bourret et al. [21] |
| Margaree | MGR | 21 | -61.1 | 46.43 | Gulf of St. Lawrence | Moore et al. [26],  Bradbury et al. [36],  Bourret et al. [21] |
| Cap Chat | CC | 20 | -66.83 | 49.08 | Gulf of St. Lawrence | Moore et al. [26],  Bradbury et al. [36],  Bourret et al. [21] |
| Jacquet | JT | 20 | -66.02 | 47.92 | Gulf of St. Lawrence | Moore et al. [26],  Bradbury et al. [36],  Bourret et al. [21] |
| North River NS | NRH | 22 | -63.31 | 45.38 | Gulf of St. Lawrence | Moore et al. [26],  Bradbury et al. [36],  Bourret et al. [21] |
| Nashwaak | NSH | 20 | -66.62 | 45.96 | St. John River | Moore et al. [26],  Bradbury et al. [36],  Bourret et al. [21] |
| Tobique | TOB | 20 | -67.7 | 46.77 | St. John River | Moore et al. [26],  Bradbury et al. [36],  Bourret et al. [21] |
| Stewiacke | STW | 27 | -63.38 | 45.14 | Bay of Fundy | Moore et al. [26],  Bradbury et al. [36],  Bourret et al. [21] |
| Big Salmon | BSR | 20 | -65.41 | 45.42 | Bay of Fundy | Moore et al. [26],  Bradbury et al. [36],  Bourret et al. [21] |
| Gaspereau | GAK | 20 | -64.27 | 45.1 | Bay of Fundy | Moore et al. [26],  Bradbury et al. [36],  Bourret et al. [21] |
| LaHave | LAH | 22 | -64.5 | 44.37 | Nova Scotia | Moore et al. [26],  Bradbury et al. [36],  Bourret et al. [21] |
| Medway | MED | 10 | -64.63 | 44.13 | Nova Scotia | Moore et al. [26],  Bradbury et al. [36],  Bourret et al. [21] |
| St Mary's | SMA | 20 | -61.95 | 45.08 | Nova Scotia | Moore et al. [26],  Bradbury et al. [36],  Bourret et al. [21] |
| Saint-Jean | SJQ2 | 25 | -64.43 | 48.77 | Gaspésie | Moore et al. [26],  Bradbury et al. [36],  Bourret et al. [21] |
| Sainte-Anne | SA | 25 | -66.5 | 49.12 | Gaspésie | Moore et al. [26],  Bradbury et al. [36],  Bourret et al. [21] |
| Grande Cascapedia | CS | 25 | -65.9 | 48.2 | Gaspésie | Moore et al. [26],  Bradbury et al. [36],  Bourret et al. [21] |
| Matane | MT | 25 | -67.53 | 48.85 | Gaspésie | Moore et al. [26],  Bradbury et al. [36],  Bourret et al. [21] |
| Matapedia | MAP | 25 | -66.95 | 47.97 | Gaspésie | Moore et al. [26],  Bradbury et al. [36],  Bourret et al. [21] |
| Trinite | TRI | 25 | -67.3 | 49.42 | North Shore St. Lawrence Upper | Moore et al. [26],  Bradbury et al. [36],  Bourret et al. [21] |
| Natashquan | NAT | 25 | -61.8 | 50.12 | North Shore St. Lawrence Upper | Moore et al. [26],  Bradbury et al. [36],  Bourret et al. [21] |
| Moisie | MOI | 25 | -66.07 | 50.2 | North Shore St. Lawrence Upper | Moore et al. [26],  Bradbury et al. [36],  Bourret et al. [21] |
| Saint Jean North Shore | SJQ8 | 25 | -64.33 | 50.28 | North Shore St. Lawrence Upper | Moore et al. [26],  Bradbury et al. [36],  Bourret et al. [21] |
| Musquaro | MUQ | 25 | -61.07 | 50.2 | North Shore St. Lawrence Lower | Moore et al. [26],  Bradbury et al. [36],  Bourret et al. [21] |
| Etamamiou | ET | 25 | -59.97 | 50.27 | North Shore St. Lawrence Lower | Moore et al. [26],  Bradbury et al. [36],  Bourret et al. [21] |
| Gros Mécatina | MEC | 25 | -59.08 | 50.77 | North Shore St. Lawrence Lower | Moore et al. [26],  Bradbury et al. [36],  Bourret et al. [21] |
| Du Gouffre | DG | 25 | -70.48 | 47.43 | Southern Quebec | Moore et al. [26],  Bradbury et al. [36],  Bourret et al. [21] |
| Malbaie | ML | 25 | -70.13 | 47.65 | Southern Quebec | Moore et al. [26],  Bradbury et al. [36],  Bourret et al. [21] |
| Sainte-Marguerite | SM | 25 | -69.93 | 48.25 | Southern Quebec | Moore et al. [26],  Bradbury et al. [36],  Bourret et al. [21] |
| Chaloupe | CHA | 23 | -62.53 | 49.13 | Anticosti | Moore et al. [26],  Bradbury et al. [36],  Bourret et al. [21] |
| Jupiter | JU | 25 | -63.58 | 49.47 | Anticosti | Moore et al. [26],  Bradbury et al. [36],  Bourret et al. [21] |
| Aux Saumons | SU | 24 | -62.23 | 49.42 | Anticosti | Moore et al. [26],  Bradbury et al. [36],  Bourret et al. [21] |
| Koksoak | KO | 25 | -68.17 | 58.53 | Ungava | Moore et al. [26],  Bradbury et al. [36],  Bourret et al. [21] |
| George | GE | 18 | -66.17 | 58.82 | Ungava | Moore et al. [26],  Bradbury et al. [36],  Bourret et al. [21] |
| Aux Feuilles | AF | 25 | -70.07 | 58.77 | Ungava | Moore et al. [26],  Bradbury et al. [36],  Bourret et al. [21] |
| Napetipi | NAP | 25 | -58.05 | 51.3 | Coastal Labrador | Moore et al. [26],  Bradbury et al. [36],  Bourret et al. [21] |
| Vieux Fort | VF | 25 | -58.02 | 51.32 | Coastal Labrador | Moore et al. [26],  Bradbury et al. [36],  Bourret et al. [21] |
| Forteau | FOR | 35 | -56.94 | 51.48 | Coastal Labrador | Moore et al. [26],  Bradbury et al. [36],  Bourret et al. [21] |
| L'anse au Loup | LL | 22 | -56.82 | 51.53 | Coastal Labrador | Moore et al. [26],  Bradbury et al. [36],  Bourret et al. [21] |
| St Paul | STP | 25 | -57.7 | 51.47 | Coastal Labrador | Moore et al. [26],  Bradbury et al. [36],  Bourret et al. [21] |
| Sand Hill | SH | 20 | -56.35 | 53.57 | Coastal Labrador | Sylvester et al. [38] |
| Eagle | EA | 22 | -57.47 | 53.53 | Coastal Labrador | Sylvester et al. [38] |
| Paradise | PA | 20 | -57.25 | 53.42 | Coastal Labrador | Sylvester et al. [38] |
| Southwest Brook | SW | 25 | -57.23 | 53.42 | Coastal Labrador | Sylvester et al. [38] |
| Hunt | HU | 20 | -60.67 | 55.57 | Coastal Labrador | Sylvester et al. [38] |
| Peters | PR | 21 | -60.71 | 53.34 | Lake Melville, Labrador | Sylvester et al. [38] |
| Susan | SR | 22 | -61.04 | 53.74 | Lake Melville, Labrador | Sylvester et al. [38] |
| Crooked | CR | 21 | -60.83 | 53.87 | Lake Melville, Labrador | Sylvester et al. [38] |
| Red Wine | RW | 22 | -60.99 | 53.93 | Lake Melville, Labrador | Sylvester et al. [38] |
| Kenamu | KE | 22 | -59.91 | 53.48 | Lake Melville, Labrador | Sylvester et al. [38] |
| Traverspine | TR | 22 | -60.28 | 53.28 | Lake Melville, Labrador | Sylvester et al. [38] |
| Caroline | CL | 20 | -60.42 | 53.25 | Lake Melville, Labrador | Sylvester et al. [38] |
| Cape Caribou | CB | 21 | -60.42 | 53.62 | Lake Melville, Labrador | Sylvester et al. [38] |
| Main Brook | MB | 21 | -57.87 | 54.24 | Lake Melville, Labrador | Sylvester et al. [38] |
| Sebaskachu | SK | 22 | -60.14 | 53.79 | Lake Melville, Labrador | Sylvester et al. [38] |
| Mulligan | MU | 21 | -60.09 | 53.87 | Lake Melville, Labrador | Sylvester et al. [38] |
| La Poile | LPR | 20 | -58.32 | 47.8 | Newfoundland | Moore et al. [26],  Bradbury et al. [36],  Bourret et al. [21] |
| Grey | GRR | 20 | -57.01 | 47.68 | Newfoundland | Moore et al. [26],  Bradbury et al. [36],  Bourret et al. [21] |
| St. Genevieve | SGR | 20 | -56.8 | 51.14 | Newfoundland | Moore et al. [26],  Bradbury et al. [36],  Bourret et al. [21] |
| Western Arm Brook | WAB | 20 | -56.76 | 51.19 | Newfoundland | Moore et al. [26],  Bradbury et al. [36],  Bourret et al. [21] |
| Conne | CNR | 21 | -55.7 | 47.91 | Newfoundland | Moore et al. [26],  Bradbury et al. [36],  Bourret et al. [21] |
| Garnish | GAR | 20 | -55.35 | 47.23 | Newfoundland | Moore et al. [26],  Bradbury et al. [36],  Bourret et al. [21] |
| Campbellton | CMP | 20 | -54.92 | 49.28 | Newfoundland | Moore et al. [26],  Bradbury et al. [36],  Bourret et al. [21] |
| Long Harbour | LHR | 20 | -54.94 | 47.82 | Newfoundland | Moore et al. [26],  Bradbury et al. [36],  Bourret et al. [21] |
| Bay du Nord | BDN | 18 | -55.44 | 47.73 | Newfoundland | Moore et al. [26],  Bradbury et al. [36],  Bourret et al. [21] |
| Northeast Brook Trepassey | NBT | 20 | -53.35 | 46.77 | Newfoundland | Moore et al. [26],  Bradbury et al. [36],  Bourret et al. [21] |
| Northeast River Placentia | NPR | 20 | -53.9 | 47.26 | Avalon | Moore et al. [26],  Bradbury et al. [36],  Bourret et al. [21] |
| Rocky | RKR | 40 | -53.57 | 47.22 | Avalon | Moore et al. [26],  Bradbury et al. [36],  Bourret et al. [21] |
| Salmonier | LSR | 19 | -53.45 | 47.17 | Avalon | Moore et al. [26],  Bradbury et al. [36],  Bourret et al. [21] |
| Biscay Bay | BSB | 20 | -53.28 | 46.79 | Avalon | Moore et al. [26],  Bradbury et al. [36],  Bourret et al. [21] |
| Narraguagus | NAR | 25 | -67.92 | 44.6 | Maine | Bourret et al. [21] |
| Tuloma | TUL | 40 | 33 | 68.88 | Barents-White | Bourret et al. [21] |
| Tana | TAN | 29 | 28.38 | 70.5 | Barents-White | Bourret et al. [21] |
| Emtsa | EMT | 40 | 40.48 | 64.57 | Barents-White | Bourret et al. [21] |
| Lebyazhya | LEB | 40 | 41.28 | 66.95 | Barents-White | Bourret et al. [21] |
| Yapoma | YAP | 40 | 36.85 | 66.27 | Barents-White | Bourret et al. [21] |
| Pongoma | PON | 40 | 34.63 | 65 | Barents-White | Bourret et al. [21] |
| Suma | SUM | 40 | 35.4 | 64.28 | Barents-White | Bourret et al. [21] |
| Numedalslagen | NUM | 43 | 10.05 | 59.04 | Norway | Bourret et al. [21] |
| Gaula | GAU | 43 | 10.23 | 63.34 | Norway | Bourret et al. [21] |
| Lærdalselva | LAR | 25 | 7.47 | 61.1 | Norway | Bourret et al. [21] |
| Moy | MOY | 40 | -9.15 | 54.1 | Ireland | Bourret et al. [21] |
| Blackwater | BLW | 40 | -7.84 | 51.94 | Ireland | Bourret et al. [21] |
| Loire | LOI | 39 | -2.18 | 47.27 | France | Bourret et al. [21] |
| Cares | CAR | 40 | -4.51 | 43.39 | Spain | Bourret et al. [21] |
| Piguena | PIG | 20 | -6.08 | 43.57 | Spain | Bourret et al. [21] |
| Narcea | NAC | 20 | -6.08 | 43.57 | Spain | Bourret et al. [21] |
| Sela | SEL | 32 | -14.81 | 65.83 | Iceland | Bourret et al. [21] |
| Olfusa | OLF | 32 | -21.23 | 63.93 | Iceland | Bourret et al. [21] |
| Tornionjoki | TOR | 40 | 24.15 | 65.81 | Baltic Sea | Bourret et al. [21] |
| Vindelalven | VIN | 40 | 20.32 | 63.75 | Baltic Sea | Bourret et al. [21] |
| Kunda | KUN | 40 | 26.53 | 59.52 | Baltic Sea | Bourret et al. [21] |
| Awe | AWE | 40 | -5.22 | 56.44 | UK | Gilbey et al. [37] |
| Ayr | AYR | 40 | -4.17 | 55.52 | UK | Gilbey et al. [37] |
| Beauly | BEA | 40 | -4.86 | 57.40 | UK | Gilbey et al. [37] |
| Conon | CON | 40 | -4.58 | 57.54 | UK | Gilbey et al. [37] |
| Dart | DAR | 35 | -3.63 | 50.4 | UK | Gilbey et al. [37] |
| Dee | DEE | 40 | -2.56 | 57.06 | UK | Gilbey et al. [37] |
| Deveron | DEV | 40 | -3.03 | 57.29 | UK | Gilbey et al. [37] |
| Dionard | DIO | 40 | -4.8 | 58.52 | UK | Gilbey et al. [37] |
| Don | DON | 40 | -2.85 | 57.24 | UK | Gilbey et al. [37] |
| Findhorn | FND | 40 | -3.67 | 57.45 | UK | Gilbey et al. [37] |
| Forth | FRTH | 40 | -4.04 | 56.17 | UK | Gilbey et al. [37] |
| Foyle | FOY | 40 | -7.25 | 55.05 | UK | Gilbey et al. [37] |
| Gruinard | GRU | 40 | -5.40 | 57.84 | UK | Gilbey et al. [37] |
| Carron | CARR | 40 | -4.45 | 57.89 | UK | Gilbey et al. [37] |
| Oykel - Cassley - Shin System | OYK | 40 | -4.72 | 58.08 | UK | Gilbey et al. [37] |
| Carnoch | CARN | 32 | -5.50 | 56.69 | UK | Gilbey et al. [37] |
| Moidart | MOID | 31 | -5.72 | 56.78 | UK | Gilbey et al. [37] |
| Lossie | LOS | 40 | -3.34 | 57.61 | UK | Gilbey et al. [37] |
| Nairn | NAI | 24 | -4.10 | 57.46 | UK | Gilbey et al. [37] |
| Helmsdale | HEL | 40 | -3.92 | 58.23 | UK | Gilbey et al. [37] |
| Naver | NAV | 40 | -4.35 | 58.20 | UK | Gilbey et al. [37] |
| Ness | NESS | 40 | -4.77 | 57.19 | UK | Gilbey et al. [37] |
| Nith | NITH | 40 | -3.87 | 55.43 | UK | Gilbey et al. [37] |
| North Esk | NES | 40 | -2.42 | 56.75 | UK | Gilbey et al. [37] |
| Abhainn Ghriomarstaidh | ABH | 40 | -6.83 | 58.00 | UK | Gilbey et al. [37] |
| Snizort | SNIZ | 40 | -6.30 | 57.45 | UK | Gilbey et al. [37] |
| South Esk | SESK | 40 | -2.66 | 56.72 | UK | Gilbey et al. [37] |
| Spey | SPEY | 40 | -3.27 | 57.31 | UK | Gilbey et al. [37] |
| Tay | TAY | 40 | -3.39 | 56.51 | UK | Gilbey et al. [37] |
| Ugie | UGI | 39 | -2.04 | 57.52 | UK | Gilbey et al. [37] |
| Ythan | YTH | 40 | -2.13 | 57.40 | UK | Gilbey et al. [37] |
| Aln | ALN | 40 | -1.66 | 55.40 | UK | Gilbey et al. [37] |
| Coquet | COQ | 40 | -1.75 | 55.29 | UK | Gilbey et al. [37] |
| Esk | ESK | 40 | -0.73 | 54.44 | UK | Gilbey et al. [37] |
| Ouse | OUS | 40 | -1.70 | 54.27 | UK | Gilbey et al. [37] |
| Tees | TEES | 40 | -2.04 | 54.61 | UK | Gilbey et al. [37] |
| Tweed | TWE | 37 | -1.98 | 55.75 | UK | Gilbey et al. [37] |
| Tyne | TYN | 40 | -2.31 | 54.97 | UK | Gilbey et al. [37] |
| Wear | WEAR | 40 | -1.68 | 54.67 | UK | Gilbey et al. [37] |

**Table S2.** Average annual and seasonal air temperature data, precipitation, and degree days > 0 and 10 °C from weather stations located near North American and European rivers.

| **Site** | **Code** | **Average Annual Temp** | **Annual Temp_SD** | **Winter** | **Spring** | **Summer** | **Fall** | **Annual Snowfall** | **Annual Rainfall** | **Days >0 C** | **Days >10 C** | **Degree Days >0** | **Degree Days >10** |
| --- | --- | --- | --- | --- | --- | --- | --- | --- | --- | --- | --- | --- | --- |
| **Du Gouffre** | **DG** | **4** | **2.9** | **-10** | **2.9** | **17.03** | **6.17** | **256.5** | **737** | **273** | **175.2** | **2645.3** | **803.2** |
| **Malbaie** | **ML** | **4** | **2.9** | **-10** | **2.9** | **17.03** | **6.17** | **257.5** | **737** | **273** | **175.2** | **2645.3** | **803.2** |
| **Aux Feuilles** | **AF** | **-5.4** | **1.4** | **-22.37** | **-8.63** | **10.1** | **-0.6** | **251.7** | **295.5** | **188.1** | **97.7** | **1256.9** | **182.8** |
| **Sainte-Marguerite** | **SM** | **2.8** | **3.8** | **-12.03** | **2.03** | **16.4** | **4.83** | **282.1** | **699.2** | **256.8** | **164.7** | **2437.2** | **717.4** |
| **Koksoak** | **KO** | **-5.4** | **1.4** | **-22.37** | **-8.63** | **10.1** | **-0.6** | **251.7** | **295.5** | **188.1** | **97.7** | **1256.9** | **182.8** |
| **Narraguagus** | **NAR** | **6.75** | **0** | **-5.48** | **4.83** | **17.37** | **8.58** | **158** | **1064.3** | **325** | **208.1** | **3166** | **1089** |
| **Tobique** | **TOB** | **3.5** | **1.1** | **-10.83** | **2.67** | **16.63** | **5.4** | **335** | **792.2** | **271** | **177** | **2553.7** | **766.3** |
| **Matane** | **MT** | **2** | **0.7** | **-12.83** | **0.77** | **15.57** | **4.4** | **344.6** | **687.5** | **251.6** | **161.6** | **2279.6** | **621.9** |
| **Trinite** | **TRI** | **1.7** | **0.9** | **-12.1** | **0.3** | **14.23** | **4.2** | **342.9** | **697.6** | **257.6** | **151.9** | **2057.1** | **452.8** |
| **Matapedia** | **MAP** | **3.7** | **2.8** | **-10.03** | **2.5** | **16.6** | **5.9** | **221.5** | **693.3** | **270.4** | **171** | **2529.8** | **734.7** |
| **Cap Chat** | **CC** | **3.2** | **2.8** | **-9.57** | **1.47** | **15.17** | **5.63** | **240.1** | **625.8** | **260** | **155** | **2299.1** | **576.7** |
| **Nashwaak** | **NSH** | **5.8** | **0.9** | **-7.4** | **4.63** | **18.13** | **7.87** | **214** | **885.9** | **292.6** | **196.1** | **2993** | **977** |
| **Sainte-Anne** | **SA** | **3.2** | **2.8** | **-9.57** | **1.47** | **15.17** | **5.63** | **240.1** | **625.8** | **260** | **155** | **2299.1** | **576.7** |
| **George** | **GE** | **-5.4** | **1.4** | **-22.37** | **-8.63** | **10.1** | **-0.6** | **251.7** | **295.5** | **188.1** | **97.7** | **1256.9** | **182.8** |
| **Moisie** | **MOI** | **1** | **1** | **-13.1333** | **-0.13333** | **13.8** | **3.533333** | **384.6** | **747.5** | **250** | **143.4** | **1959.5** | **408** |
| **Jacquet** | **JT** | **3.7** | **2.8** | **-10.03** | **2.5** | **16.6** | **5.9** | **220.5** | **693.3** | **270.4** | **171** | **2529.8** | **734.7** |
| **Grande Cascapedia** | **CS** | **1.9** | **1.9** | **-11.97** | **0.87** | **14.83** | **3.97** | **363.3** | **844.4** | **252** | **158.4** | **2165.2** | **541** |
| **Miramichi** | **MIR** | **4.9** | **0.7** | **-8.63** | **3.27** | **17.77** | **7.03** | **291.4** | **793.9** | **287.8** | **186.8** | **2789.7** | **912.1** |
| **Big Salmon** | **BSR** | **6.4** | **2** | **-4.6** | **4.33** | **17.23** | **8.73** | **172.2** | **1188.3** | **312.6** | **204.8** | **2955.4** | **890.3** |
| **Medway** | **MED** | **7.1** | **1.8** | **-3.73** | **5** | **17.83** | **9.17** | **199.4** | **1336.3** | **320.2** | **211.4** | **3105.4** | **978.8** |
| **LaHave** | **LAH** | **7.1** | **1.8** | **-3.73** | **5** | **17.83** | **9.17** | **199.4** | **1336.3** | **320.2** | **211.4** | **3105.4** | **978.8** |
| **Saint-Jean (Gaspésie)** | **SJQ2** | **3.6** | **1** | **-8.13** | **1.33** | **14.83** | **6.27** | **314.6** | **881** | **264.4** | **146.9** | **2258.8** | **542.5** |
| **Saint Jean North Shore** | **SJQ8** | **1.6** | **1.2** | **-11.3** | **0.13** | **13.33** | **4.27** | **255** | **839.1** | **256.8** | **144.9** | **1949.1** | **364.4** |
| **Gaspereau** | **GAK** | **7.1** | **1.1** | **-4.27** | **5.1** | **18.17** | **9.33** | **263** | **930.8** | **306.9** | **203** | **3161.8** | **1022.6** |
| **Jupiter** | **JU** | **1.6** | **1.2** | **-11.3** | **0.13** | **13.33** | **4.27** | **255** | **839.1** | **256.8** | **144.9** | **1949.1** | **364.4** |
| **Stewiacke** | **STW** | **6.4** | **2** | **-4.6** | **4.33** | **17.23** | **8.73** | **172.2** | **1188.3** | **312.6** | **204.8** | **2955.4** | **890.3** |
| **North River NS** | **NRH** | **6.4** | **2** | **-4.6** | **4.33** | **17.23** | **8.73** | **172.2** | **1188.3** | **312.6** | **204.8** | **2955.4** | **890.3** |
| **Chaloupe** | **CHA** | **1.6** | **1.2** | **-11.3** | **0.13** | **13.33** | **4.27** | **255** | **839.1** | **256.8** | **144.9** | **1949.1** | **364.4** |
| **Cross** | **CRO** | **5.7** | **2.8** | **-6.13** | **3.2** | **17.5** | **8.37** | **277.9** | **809.1** | **280.4** | **176** | **2808.7** | **882.4** |
| **Aux Saumons** | **SU** | **1.6** | **1.2** | **-11.3** | **0.13** | **13.33** | **4.27** | **255** | **839.1** | **256.8** | **144.9** | **1949.1** | **364.4** |
| **Antigonish West** | **ANW** | **6** | **2.1** | **-5.07** | **3.57** | **16.87** | **8.47** | **190.7** | **1124.4** | **300.9** | **192.9** | **2840.4** | **843** |
| **St Mary's (NS)** | **SMA** | **6** | **2.1** | **-5.07** | **3.57** | **16.87** | **8.47** | **190.7** | **1124.4** | **300.9** | **192.9** | **2840.4** | **843** |
| **Natashquan** | **NAT** | **1.2** | **2.9** | **-11.73** | **-0.63** | **13.07** | **4.1** | **355.4** | **766.5** | **257.2** | **140.3** | **1888.6** | **347.2** |
| **Margaree** | **MGR** | **5.8** | **2** | **-4.57** | **2.63** | **16.43** | **8.6** | **416.8** | **1519** | **292** | **172.7** | **2715.7** | **793** |
| **Musquaro** | **MUQ** | **1.2** | **2.9** | **-11.73** | **-0.63** | **13.07** | **4.1** | **357.4** | **766.5** | **257.2** | **140.3** | **1888.6** | **347.2** |
| **Susan River** | **SR** | **0** | **1.3** | **-15.27** | **-1.67** | **14** | **3.07** | **428.3** | **576.3** | **239.1** | **140.7** | **1952.8** | **474.3** |
| **Red Wine River** | **RW** | **0** | **1.3** | **-15.27** | **-1.67** | **14** | **3.07** | **428.3** | **576.3** | **239.1** | **140.7** | **1952.8** | **474.3** |
| **Crooked River** | **CR** | **0** | **1.3** | **-15.27** | **-1.67** | **14** | **3.07** | **428.3** | **576.3** | **239.1** | **140.7** | **1952.8** | **474.3** |
| **Peters River** | **PR** | **0** | **1.3** | **-15.27** | **-1.67** | **14** | **3.07** | **428.3** | **576.3** | **239.1** | **140.7** | **1952.8** | **474.3** |
| **Hunt River** | **HU** | **-1.3** | **2.9** | **-14.73** | **-3.63** | **10.67** | **2.5** | **411.4** | **538.8** | **219.5** | **99.5** | **1486.3** | **267.9** |
| **North** | **NRV** | **6.3** | **2.4** | **-4.23** | **3.3** | **16.83** | **9.1** | **312** | **1222.6** | **307.2** | **184.8** | **2859.7** | **838** |
| **Eskasoni** | **ESK** | **6.3** | **2.4** | **-4.23** | **3.3** | **16.83** | **9.1** | **312** | **1222.6** | **307.2** | **184.8** | **2859.7** | **838** |
| **Cape Caribou** | **CB** | **0** | **1.3** | **-15.27** | **-1.67** | **14** | **3.07** | **428.3** | **576.3** | **239.1** | **140.7** | **1952.8** | **474.3** |
| **Caroline River** | **CL** | **0** | **1.3** | **-15.27** | **-1.67** | **14** | **3.07** | **428.3** | **576.3** | **239.1** | **140.7** | **1952.8** | **474.3** |
| **Traverspine River** | **TR** | **0** | **1.3** | **-15.27** | **-1.67** | **14** | **3.07** | **428.3** | **576.3** | **239.1** | **140.7** | **1952.8** | **474.3** |
| **Sebaskachu River** | **SK** | **0** | **1.3** | **-15.27** | **-1.67** | **14** | **3.07** | **428.3** | **576.3** | **239.1** | **140.7** | **1952.8** | **474.3** |
| **Mulligan River** | **MU** | **0** | **1.3** | **-15.27** | **-1.67** | **14** | **3.07** | **428.3** | **576.3** | **239.1** | **140.7** | **1952.8** | **474.3** |
| **Etamamiou** | **ET** | **1.2** | **2.9** | **-11.73** | **-0.63** | **13.07** | **4.1** | **358.4** | **766.5** | **257.2** | **140.3** | **1888.6** | **347.2** |
| **Kenamu River** | **KE** | **0** | **1.3** | **-12.2** | **-2.4** | **11.2** | **3.57** | **464** | **616.8** | **243.9** | **115.6** | **1601.6** | **269.9** |
| **Gros Mécatina** | **MEC** | **2.4** | **1.5** | **-8.5** | **-0.03** | **12.73** | **5.17** | **434.4** | **711.8** | **258.8** | **138** | **1944.4** | **352.5** |
| **La Poile** | **LPR** | **3.7** | **2** | **-6.93** | **1.23** | **14.33** | **6.03** | **336.1** | **1159.8** | **165.9** | **167.2** | **2238.2** | **506.5** |
| **Napetipi** | **NAP** | **1.3** | **2.6** | **-9.33** | **-1.17** | **11.27** | **4.6** | **276.3** | **762.8** | **253** | **116.4** | **1657.1** | **233.7** |
| **Vieux Fort** | **VF** | **1.3** | **2.6** | **-9.33** | **-1.17** | **11.27** | **4.6** | **277.3** | **762.8** | **253** | **116.4** | **1657.1** | **233.7** |
| **Main Brook** | **MB** | **0** | **1.3** | **-15.27** | **-1.67** | **14** | **3.07** | **428.3** | **576.3** | **239.1** | **140.7** | **1952.8** | **474.3** |
| **St Paul River** | **STP** | **1.3** | **2.6** | **-9.33** | **-1.17** | **11.27** | **4.6** | **278.3** | **762.8** | **253** | **116.4** | **1657.1** | **233.7** |
| **Eagle River** | **EA** | **0** | **1.3** | **-12.2** | **-2.4** | **11.2** | **3.57** | **465** | **616.8** | **243.9** | **115.6** | **1601.6** | **269.9** |
| **Paradise River** | **PA** | **0** | **1.3** | **-12.2** | **-2.4** | **11.2** | **3.57** | **463** | **616.8** | **243.9** | **115.6** | **1601.6** | **269.9** |
| **Southwest Brook** | **SW** | **0** | **1.3** | **-12.2** | **-2.4** | **11.2** | **3.57** | **462** | **616.8** | **243.9** | **115.6** | **1601.6** | **269.9** |
| **Grey** | **GRR** | **4.6** | **2.4** | **-6.07** | **2.03** | **15.27** | **7.03** | **222.7** | **727.3** | **287.2** | **163.8** | **2438.3** | **623.7** |
| **Forteau River** | **FOR** | **1.3** | **2.6** | **-9.33** | **-1.17** | **11.27** | **4.6** | **279.3** | **762.8** | **253** | **116.4** | **1657.1** | **233.7** |
| **L'anse au Loup River** | **LL** | **1.3** | **2.6** | **-9.33** | **-1.17** | **11.27** | **4.6** | **280.3** | **762.8** | **253** | **116.4** | **1657.1** | **233.7** |
| **St. Genevieve** | **SGR** | **2** | **2.4** | **-9.47** | **-0.53** | **12.97** | **5** | **515** | **708.8** | **259.5** | **132.2** | **1930.2** | **392.1** |
| **Western Arm** | **WAB** | **2** | **2.4** | **-9.47** | **-0.53** | **12.97** | **5** | **516** | **708.8** | **259.5** | **132.2** | **1930.2** | **392.1** |
| **Sand Hill River** | **SH** | **0** | **1.3** | **-15.27** | **-1.67** | **14** | **3.07** | **428.3** | **576.3** | **239.1** | **140.7** | **1952.8** | **474.3** |
| **Conne** | **CNR** | **4.9** | **3** | **-5.2** | **2.6** | **14.87** | **7.4** | **275.2** | **1317.9** | **306.3** | **177.4** | **2477.8** | **587.3** |
| **Bay du Nord** | **BDN** | **4.9** | **3** | **-5.2** | **2.6** | **14.87** | **7.4** | **275.2** | **1317.9** | **306.3** | **177.4** | **2477.8** | **587.3** |
| **Garnish** | **GAR** | **5.4** | **3.4** | **-2.8** | **2.37** | **13.87** | **8.27** | **189.2** | **1232.3** | **312.1** | **165.2** | **2404.7** | **503.1** |
| **Long Harbour** | **LHR** | **4.9** | **3** | **-5.2** | **2.6** | **14.87** | **7.4** | **275.2** | **1317.9** | **306.3** | **177.4** | **2477.8** | **587.3** |
| **Campbellton** | **CMP** | **4.7** | **2.4** | **-6.3** | **2.37** | **15.7** | **6.93** | **299.3** | **840.3** | **286.7** | **165.9** | **2508.5** | **702** |
| **Northeast River Placentia** | **NPR** | **5.1** | **1.8** | **-4.17** | **2.57** | **14.53** | **7.67** | **205** | **1302** | **310** | **176** | **2449.2** | **557.4** |
| **Rocky River** | **RKR** | **4.5** | **1.9** | **-4.9** | **1.9** | **13.93** | **7** | **228.9** | **1235.8** | **297.4** | **163.2** | **2286.6** | **495.6** |
| **Salmonier** | **LSR** | **6.3** | **2** | **-2.2** | **3.3** | **15.17** | **8.87** | **174.4** | **1015** | **319.8** | **179.1** | **2680.2** | **668.3** |
| **Northeast Brook Trepassey** | **NBT** | **4.9** | **1.6** | **-3.17** | **2.03** | **13.1** | **7.47** | **171.8** | **1411.4** | **303.2** | **153.6** | **2233.8** | **420.6** |
| **Biscay Bay River** | **BSB** | **4.9** | **1.6** | **-3.17** | **2.03** | **13.1** | **7.47** | **171.8** | **1411.4** | **303.2** | **153.6** | **2233.8** | **420.6** |
| **Olfusa** | **OLF** | **4.4** | **-0.17** | **3.33** | **10.17** | **4.33** | **791** |  |  |  |  |  |  |
| **Sela** | **SEL** | **3.5** | **-2** | **2.17** | **10.5** | **3.33** | **488** |  |  |  |  |  |  |
| **Moy** | **MOY** | **9.6** | **5.5** | **8.17** | **14.5** | **10.5** | **729** |  |  |  |  |  |  |
| **Blackwater** | **BLW** | **9.3** | **4.83** | **8** | **14.33** | **9.67** | **566** |  |  |  |  |  |  |
| **Foyle** | **FOY** | **8.9** | **4.17** | **7.83** | **14.17** | **9.5** | **860** |  |  |  |  |  |  |
| **Abhainn Ghriomarstaidh** | **ABH** | **7.9** | **4.33** | **6.83** | **12.33** | **8.67** | **1173** |  |  |  |  |  |  |
| **Snizort** | **SNIZ** | **7.9** | **4.33** | **6.83** | **12.33** | **8.67** | **1173** |  |  |  |  |  |  |
| **Narcea** | **NAC** | **13.8** | **9.5** | **12.5** | **18.5** | **15** | **969** |  |  |  |  |  |  |
| **Piguena** | **PIG** | **13.8** | **9.5** | **12.5** | **18.5** | **15** | **969** |  |  |  |  |  |  |
| **Moidart** | **MOID** | **8.2** | **3.33** | **7.33** | **13.83** | **8.83** | **991** |  |  |  |  |  |  |
| **Carnoch River** | **CARN** | **8.2** | **3.33** | **7.33** | **13.83** | **8.83** | **991** |  |  |  |  |  |  |
| **Gruinard River** | **GRU** | **8.2** | **3.33** | **7.33** | **13.83** | **8.83** | **991** |  |  |  |  |  |  |
| **Awe** | **AWE** | **8.2** | **3.33** | **7.33** | **13.83** | **8.83** | **991** |  |  |  |  |  |  |
| **Beauly** | **BEA** | **8.2** | **3.33** | **7.33** | **13.83** | **8.83** | **991** |  |  |  |  |  |  |
| **Conon** | **CON** | **8.2** | **3.33** | **7.33** | **13.83** | **8.83** | **991** |  |  |  |  |  |  |
| **Dionard** | **DIO** | **8.2** | **3.33** | **7.33** | **13.83** | **8.83** | **991** |  |  |  |  |  |  |
| **Oykel - Cassley - Shin System** | **OYK** | **8.2** | **3.33** | **7.33** | **13.83** | **8.83** | **991** |  |  |  |  |  |  |
| **Cares** | **CARES** | **13.8** | **9.5** | **12.5** | **18.5** | **15** | **969** |  |  |  |  |  |  |
| **Carron (Bonar Bridge)** | **CARR** | **8.2** | **3.33** | **7.33** | **13.83** | **8.83** | **991** |  |  |  |  |  |  |
| **Forth** | **FRTH** | **8.2** | **3.33** | **7.33** | **13.83** | **8.83** | **991** |  |  |  |  |  |  |
| **Naver** | **NAV** | **8.2** | **3.33** | **7.33** | **13.83** | **8.83** | **991** |  |  |  |  |  |  |
| **Ness** | **NESS** | **8.2** | **3.33** | **7.33** | **13.83** | **8.83** | **991** |  |  |  |  |  |  |
| **Ayr** | **AYR** | **8.2** | **3.33** | **7.33** | **13.83** | **8.83** | **991** |  |  |  |  |  |  |
| **Nairn** | **NAI** | **8.2** | **3.33** | **7.33** | **13.83** | **8.83** | **991** |  |  |  |  |  |  |
| **Helmsdale** | **HEL** | **8.2** | **3.33** | **7.33** | **13.83** | **8.83** | **991** |  |  |  |  |  |  |
| **Nith** | **NITH** | **8.2** | **3.33** | **7.33** | **13.83** | **8.83** | **991** |  |  |  |  |  |  |
| **Findhorn** | **FND** | **8.2** | **3.33** | **7.33** | **13.83** | **8.83** | **991** |  |  |  |  |  |  |
| **Dart** | **DAR** | **10.2** | **5** | **9** | **15.83** | **11** | **982** |  |  |  |  |  |  |
| **Tay** | **TAY** | **8.6** | **3.5** | **7.5** | **14.33** | **9.33** | **761** |  |  |  |  |  |  |
| **Lossie** | **LOS** | **8.6** | **3.5** | **7.5** | **14.33** | **9.33** | **761** |  |  |  |  |  |  |
| **Spey** | **SPEY** | **8.6** | **3.5** | **7.5** | **14.33** | **9.33** | **761** |  |  |  |  |  |  |
| **Deveron** | **DEV** | **8.6** | **3.5** | **7.5** | **14.33** | **9.33** | **761** |  |  |  |  |  |  |
| **Don** | **DON** | **8.6** | **3.5** | **7.5** | **14.33** | **9.33** | **761** |  |  |  |  |  |  |
| **South Esk** | **SESK** | **8.6** | **3.5** | **7.5** | **14.33** | **9.33** | **761** |  |  |  |  |  |  |
| **Dee** | **DEE** | **8.6** | **3.5** | **7.5** | **14.33** | **9.33** | **761** |  |  |  |  |  |  |
| **North Esk** | **NESK** | **8.6** | **3.5** | **7.5** | **14.33** | **9.33** | **761** |  |  |  |  |  |  |
| **Tyne** | **TYN** | **8.9** | **3.5** | **7.83** | **15** | **10** | **840** |  |  |  |  |  |  |
| **Loire** | **LOI** | **12** | **6.33** | **10.67** | **18.17** | **12.83** | **736** |  |  |  |  |  |  |
| **Ythan** | **YTH** | **8.6** | **3.5** | **7.5** | **14.33** | **9.33** | **761** |  |  |  |  |  |  |
| **Tweed** | **TWE** | **8.5** | **3.5** | **7.5** | **14** | **9** | **668** |  |  |  |  |  |  |
| **Coquet** | **COQ** | **8.5** | **3.5** | **7.5** | **14** | **9** | **668** |  |  |  |  |  |  |
| **Ugie** | **UGI** | **8.5** | **3.5** | **7.5** | **14** | **9** | **668** |  |  |  |  |  |  |
| **Aln** | **ALN** | **8.5** | **3.5** | **7.5** | **14** | **9** | **668** |  |  |  |  |  |  |
| **Ouse** | **OUS** | **8.5** | **3.5** | **7.5** | **14** | **9** | **668** |  |  |  |  |  |  |
| **Wear** | **WEAR** | **8.5** | **3.5** | **7.5** | **14** | **9** | **668** |  |  |  |  |  |  |
| **Tees** | **TEE** | **8.5** | **3.5** | **7.5** | **14** | **9** | **668** |  |  |  |  |  |  |
| **Esk** | **EESK** | **8.5** | **3.5** | **7.5** | **14** | **9** | **668** |  |  |  |  |  |  |
| **Lærdalselva** | **LAR** | **7.9** | **2.17** | **6.67** | **14.33** | **8.5** | **2250** |  |  |  |  |  |  |
| **Numedalslagen** | **NUM** | **6.2** | **-3.5** | **5.5** | **16.33** | **6.33** | **763** |  |  |  |  |  |  |
| **Gaula** | **GAU** | **5** | **-2.67** | **7.33** | **17.67** | **5.67** | **892** |  |  |  |  |  |  |
| **Vindelalven** | **VIN** | **2.6** | **-8.33** | **1.17** | **13.83** | **3.5** | **601** |  |  |  |  |  |  |
| **Tornionjoki** | **TOR** | **1.9** | **-10.17** | **0.83** | **14.33** | **2.67** | **433** |  |  |  |  |  |  |
| **Kunda** | **KUN** | **5.2** | **-4** | **3.83** | **15.17** | **5.83** | **565** |  |  |  |  |  |  |
| **Tana** | **TAN** | **2.8** | **-3.67** | **0.67** | **10.83** | **3.5** | **1000** |  |  |  |  |  |  |
| **Tuloma** | **TUL** | **0.2** | **-10** | **-1.37** | **11.3** | **1.03** | **481** |  |  |  |  |  |  |
| **Pongoma** | **PON** | **1.1** | **-10.5** | **-0.57** | **13.03** | **2.33** | **465** |  |  |  |  |  |  |
| **Suma** | **SUM** | **1.1** | **-10.5** | **-0.57** | **13.03** | **2.33** | **465** |  |  |  |  |  |  |
| **Yapoma** | **YAP** | **0.2** | **-10** | **-1.37** | **11.3** | **1.03** | **481** |  |  |  |  |  |  |
| **Emtsa** | **EMT** | **0.8** | **-12.13** | **-0.3** | **13.73** | **1.7** | **558** |  |  |  |  |  |  |
| **Lebyazhya** | **LEB** | **0.2** | **-10** | **-1.37** | **11.3** | **1.03** | **481** |  |  |  |  |  |  |

**Table S3.** Analysis of molecular variance using 1773 SNPs assessing the significance and genetic differentiation among 134 North American and European rivers.

| **Source of Variation** | **d.f.** | **Sum of Squares** | **Variance Components** | **Percentage of Variation** |
| --- | --- | --- | --- | --- |
| Among continents | 1 | 566382.36 | 141.21 | 36.90 |
| Among populations within continents | 132 | 206391.79 | 21.00 | 5.75 |
| Within populations | 8001 | 1755663.05 | 219.43 | 57.35 |
| *F*_ST_=0.427; *F*_SC_=0.091; *F*_CT_=0.369 | | | | |

**Table S4.** McFadden’s pseudo-r^2^ and Akaike Information Criteria scores between clinal clustering coefficients (STRUCTURE Q-values, DAPC membership probabilities, and SPCA normalised lagged scores) and environmental variables, including latitude, distance, temperature, precipitation, and degree days separated by continent. All relationships were significant at P<0.0001.

|  |  | **Europe** |  |  | **North America** |  |
| --- | --- | --- | --- | --- | --- | --- |
| **Environmental Variable** | **DAPC** | **STRUCTURE** | **SPCA** | **DAPC** | **STRUCTURE** | **SPCA** |
| Latitude r^2^ | 0.88 | 0.78 | 0.74 | 0.96 | 0.86 | 0.84 |
| Latitude AIC | 746.05 | 1034.25 | 652.76 | 334.92 | 1025.827 | 834.28 |
| Distance r^2^ | 0.96 | 0.88 | 0.90 | 0.70 | 0.68 | 0.65 |
| Distance AIC | 259.30 | 638.03 | 431.75 | 2534.25 | 1930.76 | 1466.04 |
| Annual Temperature r^2^ | 0.94 | 0.89 | 0.90 | 0.89 | 0.69 | 0.68 |
| Annual Temperature AIC | 343.33 | 612.39 | 432.86 | 964.68 | 1893.02 | 1365.50 |
| Spring r^2^ | 0.94 | 0.86 | 0.89 | 0.99 | 0.76 | 0.72 |
| Spring AIC | 396.37 | 736.40 | 443.78 | 161.35 | 1504.31 | 1223.42 |
| Summer r^2^ | 0.28 | 0.17 | 0.23 | 0.37 | 0.48 | 0.49 |
| Summer AIC | 4254.82 | 3446.95 | 1389.67 | 5373.86 | 2978.53 | 1975.36 |
| Fall r^2^ | 0.93 | 0.88 | 0.88 | 0.72 | 0.60 | 0.61 |
| Fall AIC | 356.54 | 652.52 | 460.29 | 2351.14 | 2317.42 | 1566.04 |
| Winter r^2^ | 0.85 | 0.90 | 0.92 | 0.59 | 0.51 | 0.53 |
| Winter AIC | 908.54 | 559.83 | 395.64 | 3511.74 | 2794.77 | 1828.04 |
| Rainfall r^2^ | 0.31 | 0.28 | 0.18 | 0.66 | 0.43 | 0.43 |
| Rainfall AIC | 4057.15 | 3032.12 | 1452.17 | 2867.84 | 3187.47 | 2138.00 |
| Snowfall r^2^ |  | | | 0.27 | 0.32 | 0.38 |
| Snowfall AIC |  |  |  | 6163.797 | 3773.12 | 2326.33 |
| Degree Days > 0°C r^2^ |  |  |  | 0.56 | 0.60 | 0.60 |
| Degree Days > 0°C AIC |  |  |  | 3764.86 | 2349.87 | 1608.96 |
| Degree Days >10°C r^2^ |  |  |  | 0.33 | 0.44 | 0.46 |
| Degree Days >10°C AIC |  |  |  | 5644.92 | 3166.33 | 2071.67 |

**Table S5.** Clinal SNP names, sequences, genome location, gene name, and BLAST results for North American and European clinal outlier and neutral loci.

| SNP | Genome Location | Sequence | Identity | E-value | Putative Gene | Function |
| --- | --- | --- | --- | --- | --- | --- |
| ESTNV_26758_619 | NA | CCTGTGTGACACTCTGTGTTCGGACCCCTATGCCCTCGACCCTAACGCCGTGCCCTCCCTGGAGCAGCTGGACGGCTTCCGCCAGCGCATTGCCAGCCAGACCACAGAGAAGGAGCGGCGGCACGCTGAATTTGTGGGCATCAAGAGGCAGATCATCCTGTGCATGGACGACCTGGACCAGCTGCCAGAGACCAGCTTCGAGAAGGACGTCGTCTGCGAGGACCAGGAGGCCTTCTGCCTGTCCAAAGACAACATCGCCTCACTCAAACTCCTCCTCGGCCAACTAGAGGAGAGGAAGGCAGAGAACCAGGCGGTGTGTGAGGCTCACAGGGAGAAGATCCAGGAGCTGTGGGACAGACTGCAGGTGCCCCAGGAGGAGAGGGAGCTCTTCTCAGAACACATGGTCGCCTCCAAGAAGAAGAACCTGGATGCTTTAGAGGCAGAGGGCAGGCGACTGATGGAGCTCAAACGACTGAACATGCGAAACGTTACTGAAGCCATCCGCTCAGAGATCGCAGGGCTCTGGGAGAAATGCTTCTTCAGCAGTGATCAGCAACAGGCCTTTGTGCCCTATTTTAGCAATGATTTTACAGAGGAGCTGTTGGGGCTGCACGAAGCKGAGATCCTGAGGCTGAAGCAGCACTATGAAGAACACAAGGAGCTGTTTGAGGGGGTTCACAGCTGGGAGGAGAGCTGGAGACTCTTCCTGGAACTGGAGGAAAAAGCCACAGACCCCTCCAGGTTCACCAACAGAGGAGGGAATCTGCTCAAAGAGGAGAAACAGAAAGCAGAGCTCCACAAAAGCCTGCCCAAGCTTGAGAAGAAGCTGAAGGCTCAGATTGATGTGTGGGAGCAGGGTCAGGCCAGGGAGTTCCTGGTGAATGGACAGAAGTTCCTGCAGTTTGTAGAGGAGCAGTGGGAGCTGCACCGCATCAANAAAGAGAATGAGAAACTGGAGAGGCAACTGAAAAAGAGCAAGCAGATTGAGGTGGATATGCTGTATGGGACAGCTGTCCGGACGCCTACCAAAAGGAGATTCCTTGGCACAACTACCCCCAGCAAAACACGAAAGTTCAATGGCACCACCTCCAGCCTCTCTAGTGCCAACTCTAACAGCACCGTGCGCTCAGCCTATGGCGGAACTGTCTGCCACTCACCCTCCCGCCCCGCTCTGTCAGTCAACAAGGTCCCATCGGTACGGACCCCGGGTCGCAGTAAGCCCCCTCTTGCGGGACTGCAGGAGCGCAACAAGGAGAACATGGGCCTGGTGACCGGACTTGGAGTCCCTCTTCAGAGCGGTGGGTTTAAAACCCTGGCTAGTCCGCAGCATAACTTCAGCATTAACTCTGTCGCCAGCACTTATTCCGAGTTTGCGACGGGCTTAATCAACACAGTAATCGAATCGGTTCAATCAAGCGAGACCTCTCCCAGGCCACTAACACCAAGAGCAAGCCAGACATACTGAACTCCACCATTGCTCACCTTTGACCCCGACACCAGATGACGCGGTTGGTTGAATGACATCCTGTGTTTGTGTCCCATCAAGTCCCCACCAGCTGTCATTTTAAAGTGGATCACTATACNNNNNNNNNNNNNNNNNNNNNGTCTGCATTTCTAAGTAAATTTAAGGTAATACTGTATGTATGTGTGTATATGTTGTCGAAATAAAAAAAACCTAAAGGTCATGTTATGAAATGGTATCTATTGTAACAACCATTTTTTTTAAGCTCTAAAATAGCACGGATGACTGTTCTTTGTTTGAAAAATGGAGCAAACAGTTTTGCCTGGCATCTCGTGGTCCAAAATGGTTTTGGGGGAAAAAGGCTGGGGGGAACCCAGGGTTTTTTTTTTCCTTTAAGCCGGGTTTTCCCTATCTTATTTTACCCCAAAACAAATTGATTTATTAAATTTTTTTGCCG | 99 | 0 | regulator of cytokinesis 1 (prc1), transcript variant X1, mRNA | regulator of cytokinesis |
| GCR_cBin32622_Ctg1_88 | ssa01:117818673 | AGGAAACTGACACCTCGTCAAAAGC[A/G]GAGGCAGGCAATATCTACATGCGCA | N/A | N/A | rai14 | retinoic acid induced 14 |
| ESTNV_29406_497 | NA | TTTTTTTTTTTTTTTTCCTGGGAAAATTTTCTCAATTTATCAAACAGTGTGTATGTAACATGTTAACATGATGCTATACAGTGCTTTACGGCTTCCTAAATGAATACAAAACCATTAACGATATATTTACACAAATGAAAGTCCTTCATGATCAACATGCAAAAAAGCACTTTGTAATTTTAGGAAAATGCTGATTTACCTCAGCTGATTAATTTCACAAAACAAGGTTACCTTGTTAAATTCTTATACAATCTACATCACGTGGACTTCCACAAACAGAGGATCTAGAAATGAAATACAAACTGGCTTATGGGGCTGTAGCCTCCATTATTATGATGCGTGTTATCAGGGCTAAATTTGACTGACTGACTGATGCATTGATTTGAACAACATTCATCTCTTTAGTAAGTACACTCTTTAGTAAGTACACATAATTATGTCATTAGTTTGCCACCAGAAATGCAGAGGAAATGACCAATTACTGGACATGTCAAACMAAAAAAATATAGAGCATTTTTATAATCTGCTAAATAAAACATATCCACTTTGTCCTTTGATTTAGCCCTAGAGTCTGTACGGCCAGCAAGCCGAAACCTTCCAAAGAGGTCAAGTTCAAGTGGCACTCTCCACAACAAGGACCAGATTCTTCTTGTAGCACTCCAGACTACAGAGAGGGATCCCCGTCTTGGAGCATGAGTACCTCTTCAGATTACTGCACCCGTTTACTCCGCACCCTACCGGTGCCGGTGGAGGGGGGCACGGGGGAGCAGGGGTGGGGGCTAGCACCCCCTTGGGAAAGGAAATCCCCATTCCCTGGGCCGAGTCGCAGTAGCGGACCATGGGACACTGGGCCTGCTTGGACTTGCGTTCCCTCATGCTCTTGATCTTGGCCTTCGAGGTCTTGGTGAGCCTCTCGATGGTCTGGTTCTTGTTCTCCTCTGCCTTCTTGGCAGCCTGCAGGCGCCTCTTGCGGGCGCGCTCCTCCCTCTTCTGCATCATCTCAGCGGTCATCTCTTTCTCCTTGTAGCCCATGGGCAGCTCCAGGAGAGGCTGGTTCTGTTGTTTGTGCAGGAGGGCTTTCTGTCTGGCTGTGAGCAGTGACTCATCGACCTCCTTCTTCAGCTCTCCGTTGTCGTCCAACTCTCCTTTCTCCAGGGCGTCCAGCCACTTCTCCTCCTCGTCCACCGGCCCCCCCAGTCCTCCCAGGGAGTCAGAGTCCATGTCTGGCAGAGGAGACGGGTCCAGGTTACTGTCCTCATCAAGCCAGGCCCGGTACTGCTCGAGTGGGACGCCCTCAGAAGGCTCCTCATCGTCATCATCATCCGAGTCGTCATCATCGTCCACGATCATCAGAGGAGACGGGGAGCGGGCTACACCAGGGACCACACTGAATGTTGGCACACTTTTGGTCCCCAGCGTTTGGCCTCCCAGTTTGATCTTGAGTCGGAGCTGCG | 99 | 0 | znhi4 | Zinc finger HIT domain-containing protein 4 |
| GCR_cBin6860_Ctg1_169 | NA | ACCACCAGGGAGATAACTGGCTCTCTGCTTAGTGCCTGAGACAGACAGATATCTAAGCTGGGCCACTTTTCACAGAAAGTGAGAGCTGAGAACATGTTACTCAGGAACCTTGAATCTGAAACAAATTTCCAAAGACTSATGAAGACTTGCTAAAGTTCCTTAGTTAGGTTCATGTGATAGTGTTGTTCTGGATATTCCCGTGCCAAGAGTCTAGGTTACTTTTAGATCATGTGATTGGGTCATTGTGTCAAGTAAGAGTAAATTGGGATGTCTGAGACTACAGCCACCACCCCCATGTGTGTTGAGGTATGTAGCAGACCTGGGTTCAAAT | NA | NA | NA | NA |
| ESTNV_30653_603 | NA | GGTAACTAGTGACGACCCCATGGGGGTCTGGGAAGTGATTAACATTGTGATGAATTGACAAAAGACTTTGTCTGGGTTTTTATATTTCCACAAATATTTTATCTTTATGAGAAATGAACACATAACGTTTTGTTGGAGACATGGCTGAGAAATTTGATAACCTCGAGGAGCATCTCGAGAAGTTCGTTGAAAATATTCGACAGATGGGAATTATCGTTAGCGACTTTCAACCTAGCAGTCAAACAGGACTCAACCAAAAACTAAACTACATGATCACGGGACTGCAGGACATTGAGAAATGTCGCCAGCAGCTACATGAGATTAACGTTCCTCTCGAGGCTTTTGAATACATAGACCAGGGGCGTAACCCCCAGCTGTACACTAAGGAGTGTCTGGAAAGGGCCCTGGCCAAGAACGAGCAGGTCAAAGGAAAGATTGACACCATGACGAAATTCAAGAGCCTGCTGATCTCTGAGCTGGGGAAGGTGTTTCCAGAGGAGATGACCAAGTACAAGGCTATCCACGGAGATTACCCCCCCTCATAGTGACCCTTAACCCCCTGCTCAAAGCCACAGTGGAGTCTCACCCAGCAGGATCAATGASTCAACCTGATGACATGTATTAATATTCAGGGGATATTCATGAAAAAAGTGGGTTGTTGACGTTGAGAAATGGATTGAGAGCGTGGTAGAGGTAGTTGGAGGAGCAATTCTTTGTTTCTGATGTGGGAAGAGTAATGTGCCAAGGATGCTTTATGGATGGGTTCAAAAAAATCATAAAAGGAGCTAGAGCTGAGGATTTGATAGGAAGAACTGTCAGCCAGGAACGTTTTGCCTTTTTCGAGTTTATCTTTTAGTTGTATTGGAAATTACATTTTCTCAATCTGATATCTGGCGAACTGTTTCCTCATGCTGTGTTGAGATCCCACCTACAAGATGTACATAATGTTTATACTCATACAGAACCTTTTTTGTAAAGACTTTAAACATCATTGTACAACACTGCAGACCTGGACTGTGACTCCGTTTTGTATCAAAAGGGGTTTTGTTCAGAGGTTTGCAACCCTCATTTTGTTTTGATTTTATCTTGTTAGTTTTTGTTTGTTTTGCAAATAAATGGGTTTGTGATTGAAAGATTAGTCTGGTATTTGCTGCCATGCTAGCGCCTTGTAGACCACAATTCATGAACAGAGACTTAAAATAAAGTTCAATGTGTAAGAAGAAAAAAA | 99 | 0 | med10 | mediator complex subunit 10 |
| GCR_cBin8933_Ctg1_66 | ssa03:80103477 | CTAGAAGACTAAACTGATCCTAGATATGTCACTCCTACTCTAAGGTACTTTTTGAATACTGGCGGMCCAGGCTACTACATTGCAGACCTGGGTCACATTCAGTGAGGCAAAATGTTATGGAACATTCAGATAGAGATATATGGTGTAGAAGAGACATACTTCTCTGTCACGTAGAGTCAGCTCTATGACTTTTCTATCGGCAATGTTCATAGTTATACGCCCTCCTAAACATGACTGTTGTAAGATACTCTGAGTAAGATGGGTGCATTTTCCATTACATTTTGCTCTACAAATCAATATTATTACTACGGGACACACAGTGCCTTCGGAAAGTATTCAGA | NA | NA | NA | NA |
| GCR_cBin11610_Ctg1_200 | ssa24:121495 | TGTGTAGTGCCTCAGTGTAACTTGT[A/G]TATCCTCATAGGATGAATGCGG | NA | NA | LOC106584876 | Uncharacterized |
| BASS133_B7_H09_429 | NA | GATTAGTAGAGGCAAGTCCCTGTGA[C/T]CTCTGCACTGCTTCTCTCCACCCTC | 94 | 0 | Rargb, HoxC13ba | retinoic acid receptor gamma b; homeobox protein |
| GCR_cBin17723_Ctg1_219 | ssa25:22894154 | CAATCGGAGAAGCTCAGTGGGAGGT[A/G]ACATGTCCCTTTGCATGCTGGGACA | NA | NA | NA | NA |
| GCR_cBin3648_Ctg1_155 | ssa18:7702752 | TTACTGGGTTTAAAAGAGACAGCGC[A/G]CCCTTTTCTAAACTGTAGAGATGGC | NA | NA | ccnj | cyclin J |
| GCR_cBin27791_Ctg1_60 | NA | ACTAGTTTAGTGTATGAAGCCGACA[A/C]GTGCCAGCGTGTTCTAAAGTTCTGG | 99 | 0 | LOC106581294 | solute carrier family 12 member 9-like |
| ESTNV_36453_127 | ssa22:15775282 | GGGTTTCTTTCCATTTTTTCGCACG[C/T]GTATTTTCACTGTAGATTTCATTTG | NA | NA | LOC106582886; LOC106582887 | DEP domain-containing mTOR-interacting protein like; transcription factor MafB-like |


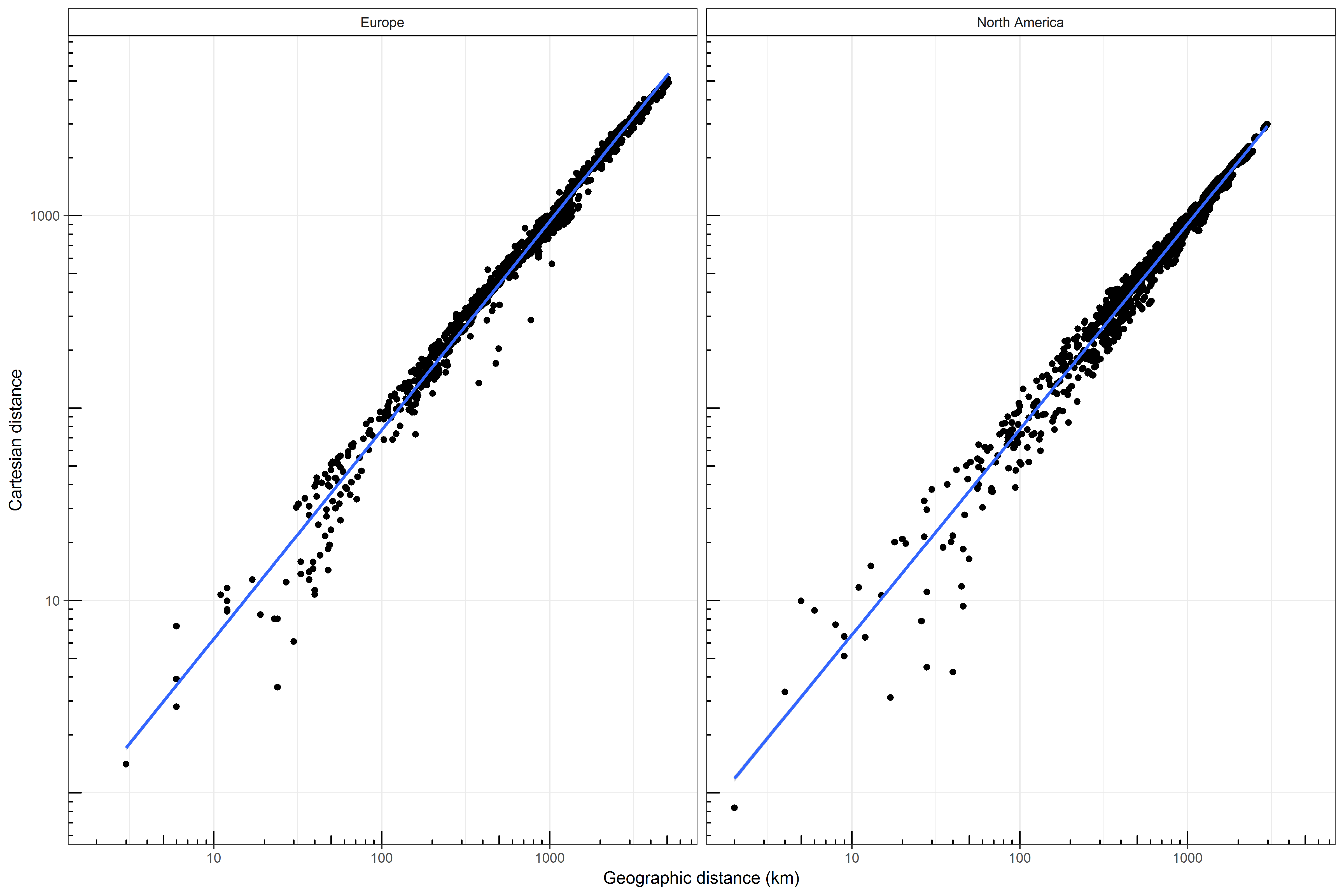


Supplementary Figure S1. A linear regression between log-scaled geographic least-cost distance versus Cartesian distance among rivers shows a strong and significant positive relationship in North America (r^2^=0.977, p<0.0001) and in Europe (r^2^=0.988, p<0.0001).


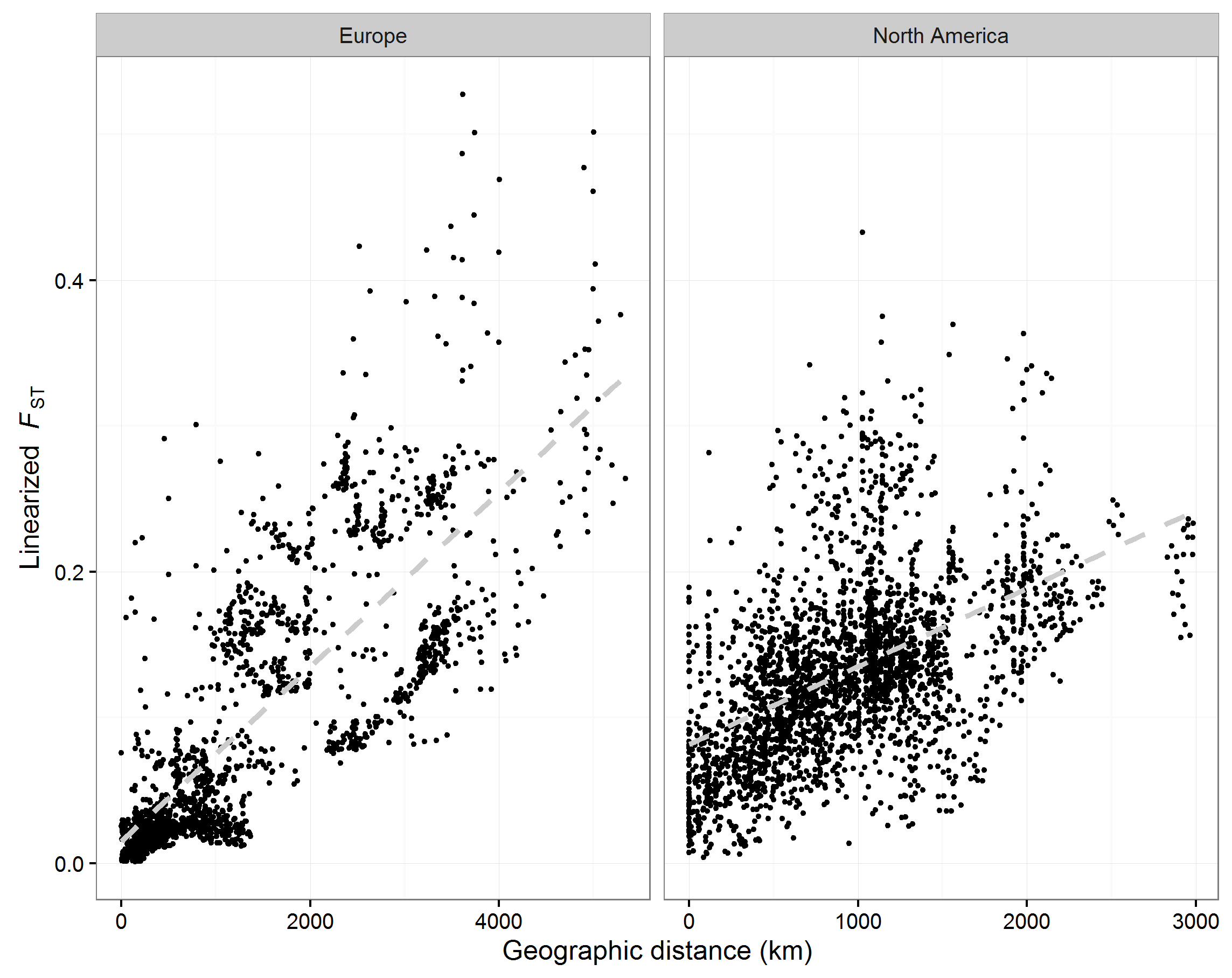


Supplementary Figure S2. Isolation-by-distance plots of European and North American rivers, showing linearized *F*_ST_ versus least-cost geographic distances.

**
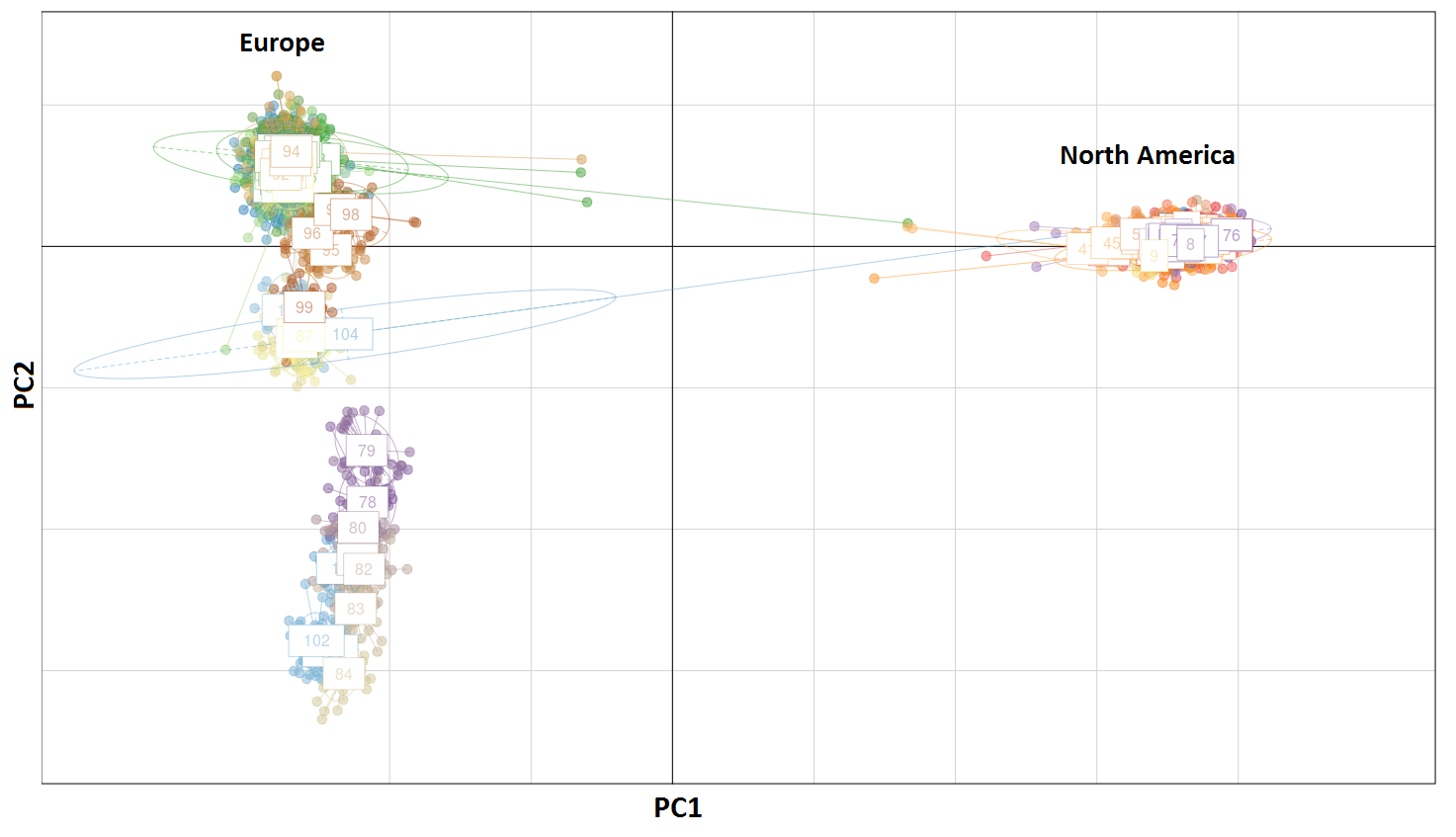
**

Supplementary Figure S3. Principal component analysis of genotype data for 134 sample sites clearly revealed distinct clustering of North American and European rivers along the first principal component axis. Europe additionally showed a high degree of structure along PC2. Population label numbers refer to the populations in Table S1.


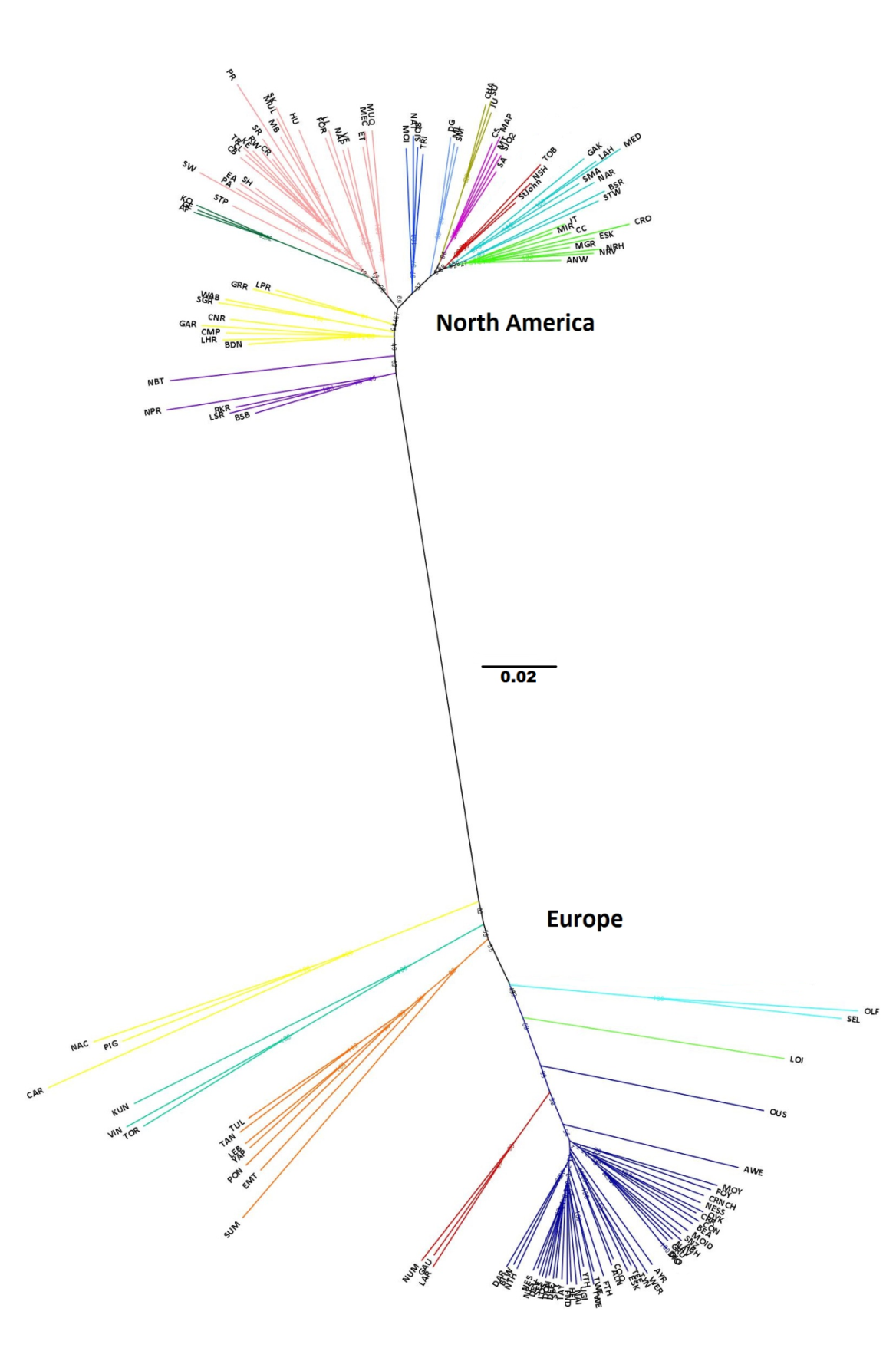


Supplementary Figure S4. Neighbour-joining tree based on Cavalli-Sforza chord distances of all populations in the present study.


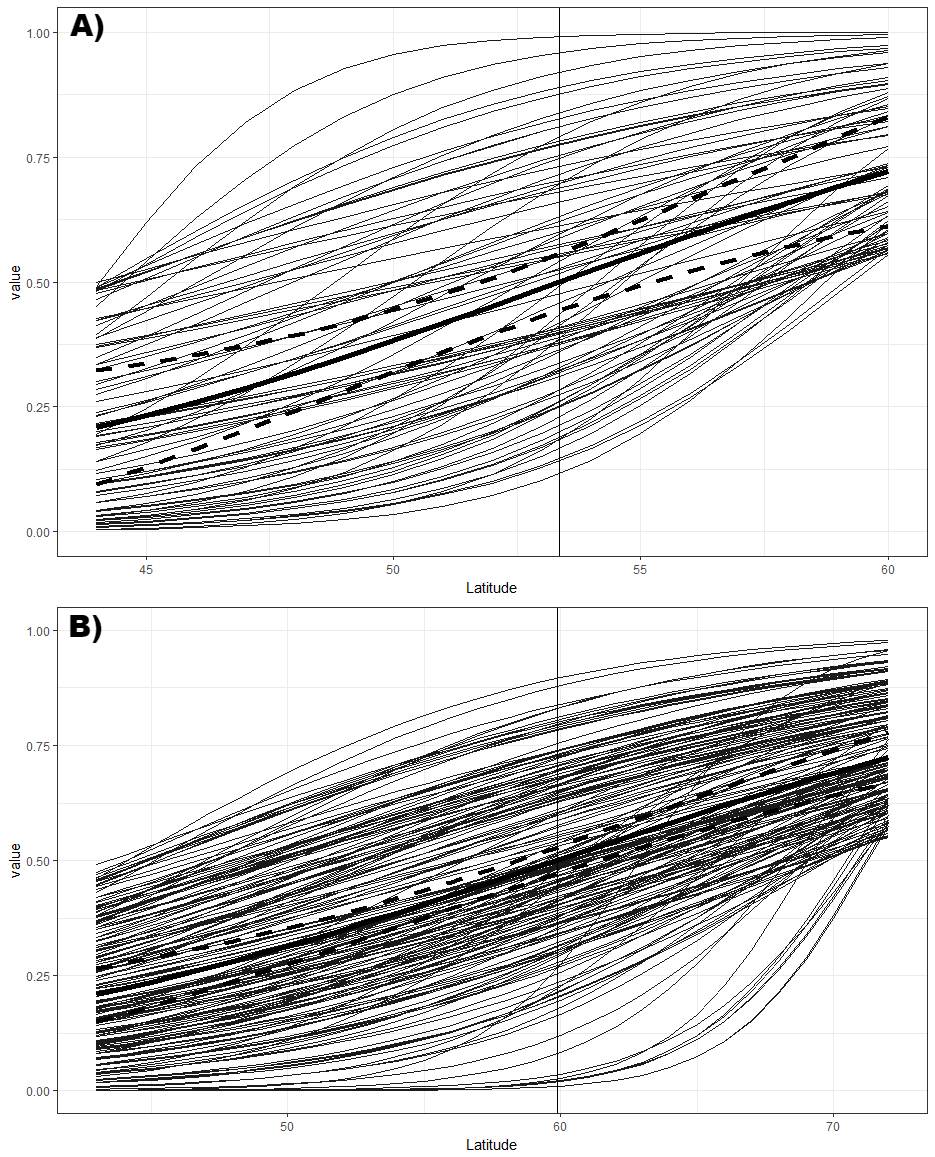


Supplementary Figure S5. Detection of clinal loci for A) North America and B) Europe. The vertical solid line indicates the predicted inflection point latitude where there is a transition between southern and northern populations, and the average cline is plotted as a thick solid line with dashed lines showing 95% confidence intervals.


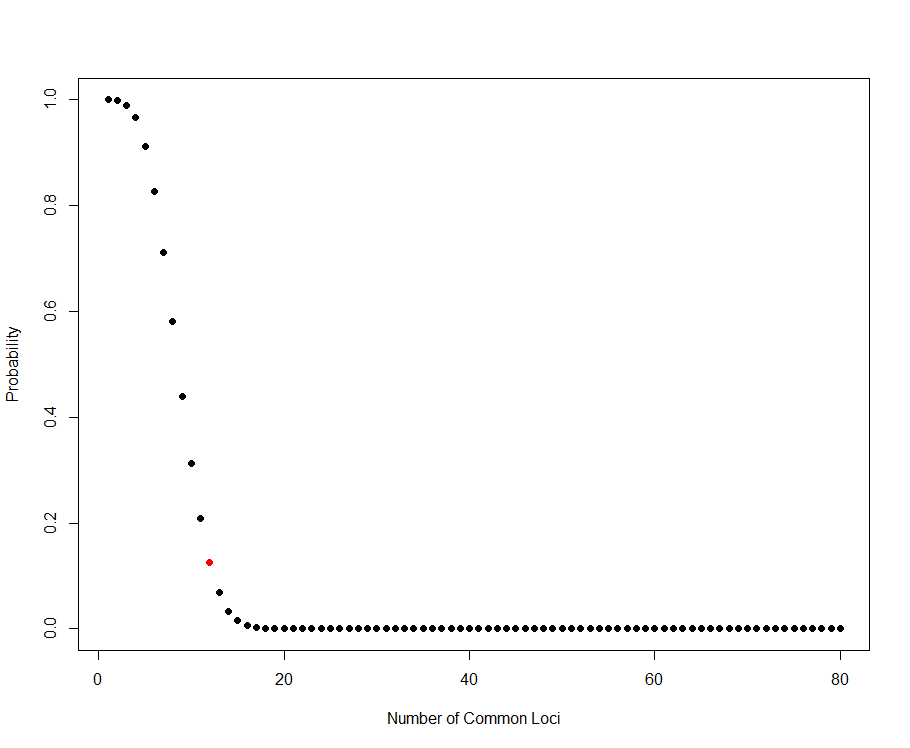


Supplementary Figure S6. A permutation test selecting 84 and 195 loci from 1773 shows a probability near 0.1 of selecting 12 loci in common. An exponential decay shows that above 12 common loci, probabilities approach zero, suggesting that this many common clinal loci is very rare.


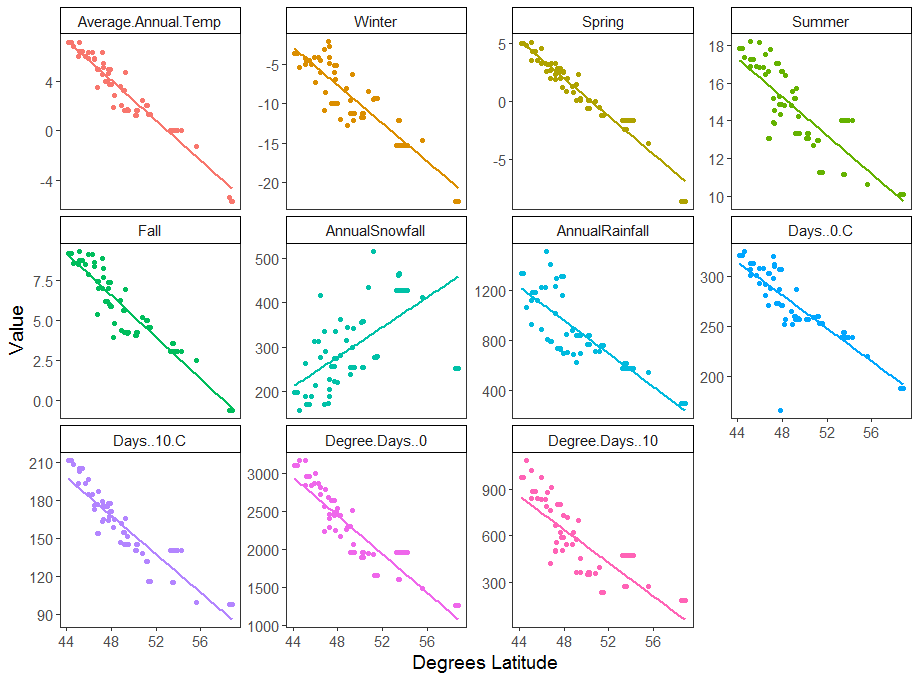


Supplementary Figure S7. Multiple regression of average annual temperature (°C), average seasonal temperatures, annual rainfall and snowfall, number of days above 0 and 10 °C, and degree days above 0 and 10 °C, versus degrees latitude for 74 North American rivers.


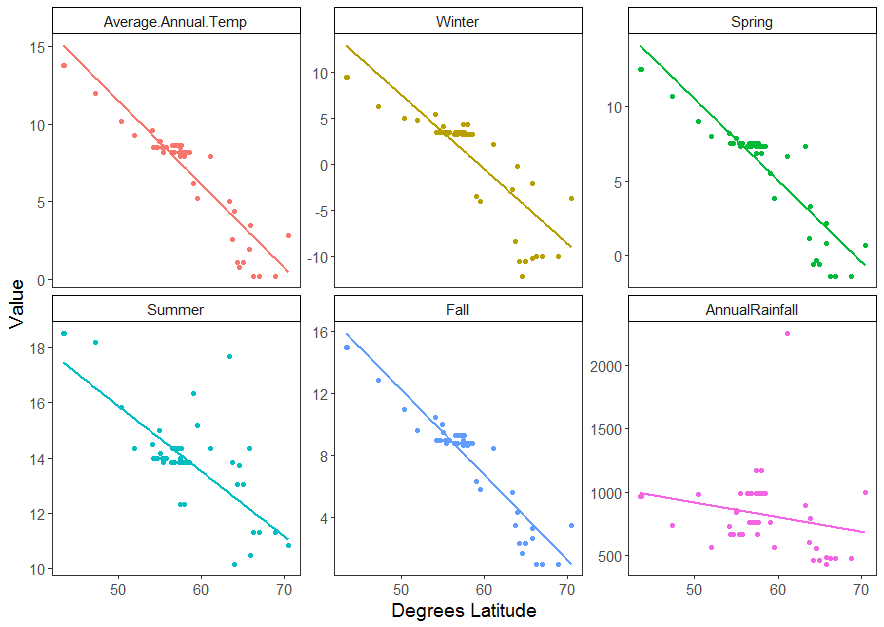


Supplementary Figure S8. Multiple regression of average annual and seasonal temperatures (°C) and annual rainfall (mm) versus degrees latitude for 60 European rivers.


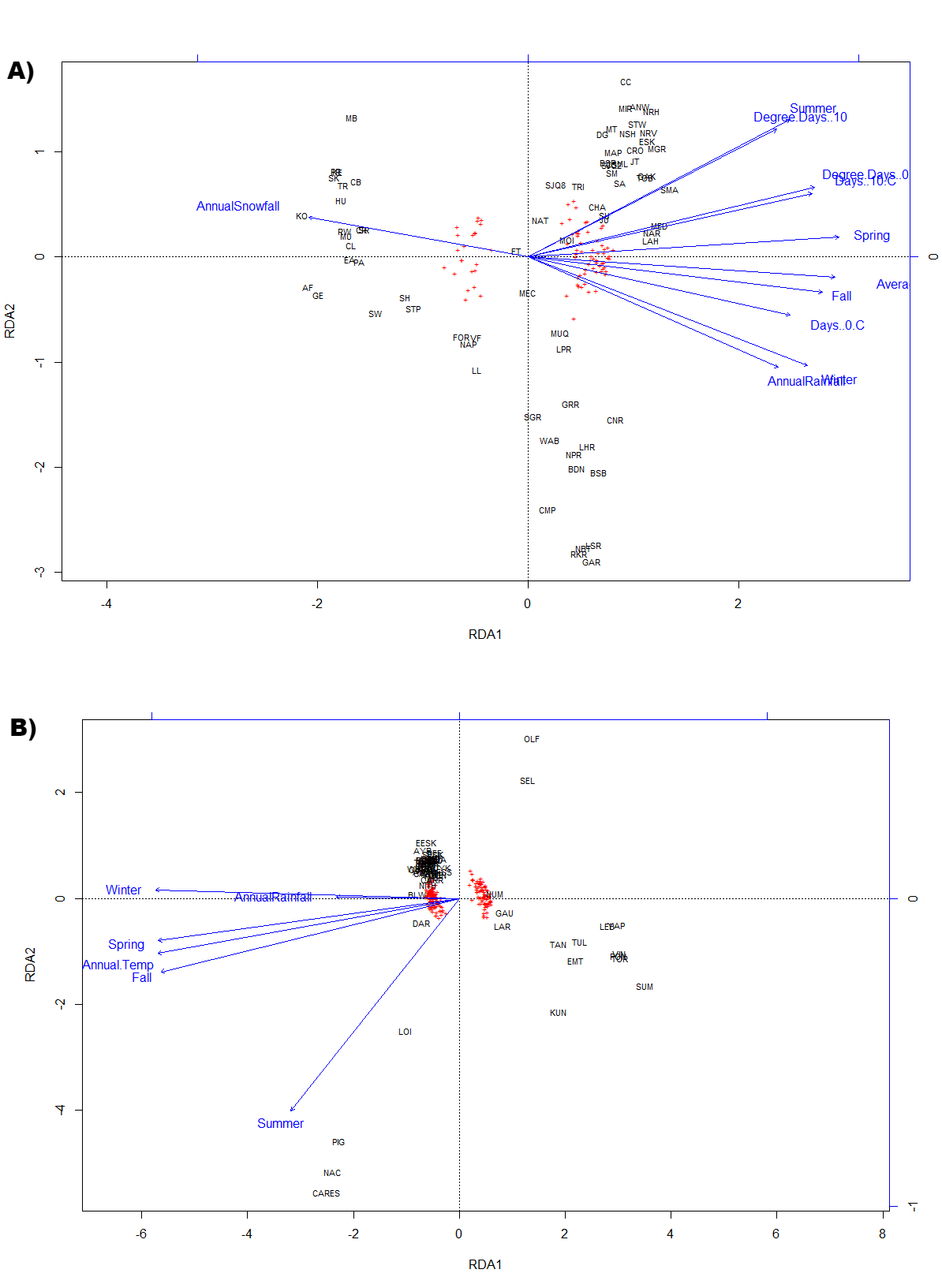


Supplementary Figure S9. Redundancy analyses on environmental variables predicting clinal allele frequency distributions for A) North America and B) Europe.


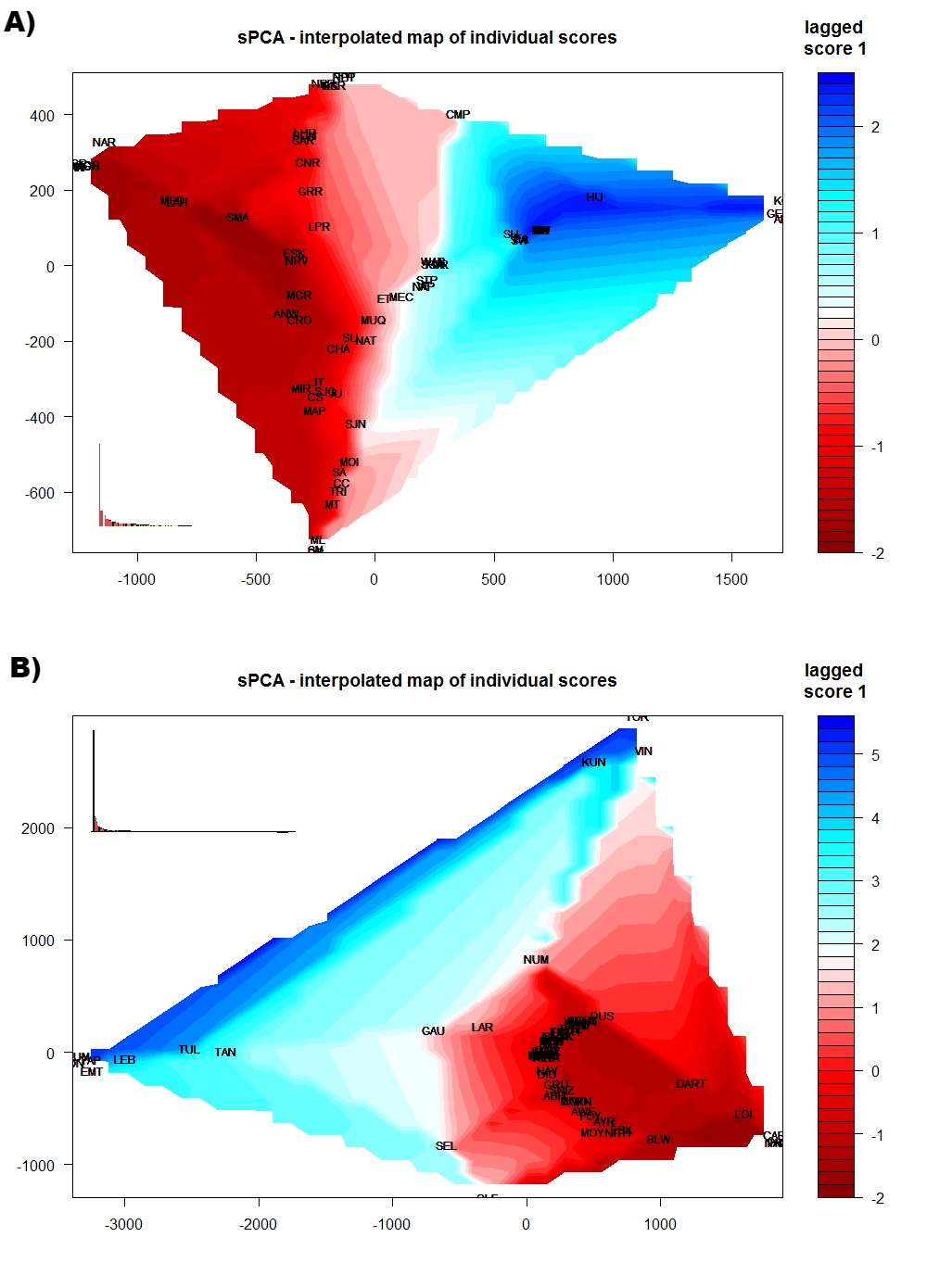


Supplementary Figure S10. Interpolated maps of individual first axis lagged scores from clinal loci based on SPCA analyses in A) North American and B) European populations. Populations are located at their Cartesian coordinates and lagged scores are plotted to show differences in spatial structure among clusters of populations. The inset bar plots show highly positive eigenvalues, which are indicative of global structure relative to little local structure.

1. Slatkin M., Voelm L. 1991 FST in a hierarchical island model. *Genetics* **127**(3), 627-629.

2. Excoffier L., Lischer H.E. 2010 Arlequin suite ver 3.5: a new series of programs to perform population genetics analyses under Linux and Windows. *Mol Ecol Resour* **10**(3), 564-567. (doi:10.1111/j.1755-0998.2010.02847.x).

3. Pante E., Simon-Bouhet B. 2013 Marmap: a package for importing, plotting and analyzing bathymetric and topographic data in R. *PLoS One* **8**(9), e73051.

4. Dray S., Dufour A.B. 2007 The ade4 package: Implementing the duality diagram for ecologists. *Journal of Statistical Software* **22**(4), 1-20.

5. Jombart T. 2008 adegenet: a R package for the multivariate analysis of genetic markers. *Bioinformatics* **24**(11), 1403-1405.

6. Jombart T., Ahmed I. 2011 adegenet 1.3-1: new tools for the analysis of genome-wide SNP data. *Bioinformatics* **27**(21), 3070-3071.

7. Cavalli-Sforza L.L., Edwards A.W. 1967 Phylogenetic analysis. Models and estimation procedures. *Am J Hum Genet* **19**(3 Pt 1), 233-257.

8. Langella O. 2015 POPULATIONS 1.2.33 software. 1999. (
